# Behavioural flexibility in an Arctic seabird using two distinct marine habitats to survive the energetic constraints of winter

**DOI:** 10.1186/s40462-022-00344-3

**Published:** 2022-11-03

**Authors:** Allison Patterson, H. Grant Gilchrist, Gregory J. Robertson, April Hedd, David A. Fifield, Kyle H. Elliott

**Affiliations:** 1 Department of Natural Resource Sciences, McGill University, Ste Anne-de-Bellevue, QC H9X 3V9 Canada; 2 Environment and Climate Change Canada, National Wildlife Research Centre, 1125 Colonel By Drive, Raven Road, Ottawa, ON K1A OH3 Canada; 3 Wildlife Research Division, Environment and Climate Change Canada, 6 Bruce Street, Mount Pearl, NL A1N 4T3 Canada

**Keywords:** Non-breeding, Nocturnal foraging, *Uria lomvia*, Labrador Sea, Daily activity rate, Biologging

## Abstract

**Background:**

Homeothermic marine animals in Polar Regions face an energetic bottleneck in winter. The challenges of short days and cold temperatures are exacerbated for flying seabirds with small body size and limited fat stores. We use biologging approaches to examine how habitat, weather, and moon illumination influence behaviour and energetics of a marine bird species, thick-billed murres (*Uria lomvia*).

**Methods:**

We used temperature-depth-light recorders to examine strategies murres use to survive winter in the Northwest Atlantic, where contrasting currents create two distinct marine habitats: cold (−0.1 ± 1.2 °C), shallower water along the Labrador Shelf and warmer (3.1 ± 0.3 °C), deep water in the Labrador Basin.

**Results:**

In the cold shelf water, murres used a high-energy strategy, with more flying and less diving each day, resulting in high daily energy expenditure and also high apparent energy intake; this strategy was most evident in early winter when day lengths were shortest. By contrast, murres in warmer basin water employed a low-energy strategy, with less time flying and more time diving under low light conditions (nautical twilight and night). In warmer basin water, murres increased diving at night when the moon was more illuminated, likely taking advantage of diel vertically migrating prey. In warmer basin water, murres dove more at night and foraging efficiency increased under negative North Atlantic Oscillation (calmer ocean conditions).

**Conclusions:**

The proximity of two distinct marine habitats in this region allows individuals from a single species to use dual (low-energy/high-energy) strategies to overcome winter energy bottlenecks.

**Supplementary Information:**

The online version contains supplementary material available at 10.1186/s40462-022-00344-3.

## Background

Animals of all kinds make seasonal and daily movements to balance energy intake and expenditure. Mobile organisms can regulate their energy balance by periodically occupying environments that increase energy intake or reduce metabolic costs [1, 2, 3, 4]. Winter strategies encompass a spectrum that includes hibernation at one end, minimizing energy output [5], and pole-to-pole migration at the opposite end, maximizing energy intake [6, 7]. Small-bodied, flying birds have limited capacity to build up energy reserves to cope with challenging environments, so they must adopt strategies that balance energy intake and energy expenditure over relatively short time scales [8, 9, 10].

During winter at high latitudes, low air and water temperatures increase energetic demands for thermoregulation at the same time as shorter day lengths limit opportunities for foraging [11, 12]. These challenges are particularly acute for diving seabirds that spend nearly all of their time on or under water, where heat loss is greater than in air, and also have to limit insulation and stored energy reserves in order to retain the ability to fly [13, 14, 15, 16]. Highly mobile species, such as seabirds, may switch among different marine habitats during winter to take advantage of more favourable conditions that increase energy intake or reduce energetic costs within their wintering areas.

Thick-billed murres (*Uria lomvia)*, hereafter murres, are a widely distributed Arctic seabird species that is declining through portions of their global range, with some declines apparently linked to wintering areas [17]. Winter has been proposed as a potential energetic bottleneck for Alcids wintering in the North Atlantic [13–15]. In Canada, murre colony sizes show synchronous patterns, indicating that conditions on shared wintering areas may be important for determining survival and subsequent breeding success [18]. Biologging devices that record aspects of animal movement and individual environment over long time-periods provide a new window for understanding the behaviour and energetics of murres during a time of year when marine birds cannot be easily observed using other methods.

Energy intake is constrained by the time available for foraging, the energy required to search for prey, and the distribution and density of prey available within the environment [17]. Because murres are wing-propelled pursuit-divers with relatively small wings adapted for swimming under water, their energetic cost of flight is five times higher than their cost of diving or swimming at the surface [19]. Generally, murres can search for prey actively, by flying to locate prey patches, or more passively, by searching under water while diving. For predators with multiple search modes, actively searching for prey should lead to increased prey capture rates, but also comes with added energetic costs associated with locomotion [17, 20]. The energy intensive (high-energy) foraging modes should be the most profitable when prey availability is high and less energy intensive (low-energy) foraging modes should be most profitable when food density is low, to reduce the total energetic costs associated with foraging [17, 20, 21]. In marine environments, prey are often patchily distributed, which may require murres to use more active search methods (e.g. flying) to locate prey patches. Examining how murres allocate time and energy between searching for prey above the water, by flying, and searching for prey below water, by diving, can provide insight into the relative prey density and distribution of different winter habitats.

Murres are visual predators, as such their time available for foraging in winter is constrained by day length and moonlight [22, 23]. Under daylight conditions murres can forage at depths down to 200 m [24], while at night maximum dive depths are less than 50 m [23] and most dives occur in the top 20 m of the water column [22]. During the breeding season murres take advantage of increased illumination from the moon to forage longer and deeper at night [22, 23]. During winter conditions that facilitate catching prey at night could allow murres to extend the time available for foraging. Because dive depth at night is limited by light availability [23], use of nocturnal foraging is likely influenced by both moon phase and weather, with higher rates of night diving when the moon is closer to full and cloud cover is low. Nocturnal foraging would be most beneficial where diel vertical migration (DVM) brings prey into the surface layers of the ocean at depths that are accessible to diving predators at night. Winter night-feeding in areas with DVM prey could be a more profitable strategy in deeper water (> 200 m), where prey can take refuge below the maximum diving depth of murres during the day. Environmental conditions that contribute to a higher biomass of DVM prey or a shallower active layer at night could promote night-feeding.

Weather can impact the behaviour and foraging of seabirds through increased energetic costs associated with increased wind or through reduced visibility and accessibility of prey [11, 25, 26]. The North Atlantic Oscillation (NAO) is an important climate pattern associated with changes in weather and ocean climate in the Northern Hemisphere [27, 28], which has a pronounced influence in both terrestrial and marine ecosystems [29]. The NAO is based on the difference in sea level pressure between a low-pressure cell located near Iceland (Icelandic Low) and a high-pressure cell located near the Azores (Azores High). A positive NAO phase (larger pressure difference) is generally associated with an intensification of the westerly winds above the North Atlantic, while a negative NAO phase (smaller pressure difference) is generally associated with weaker westerlies, as well as differences in storm track, precipitation humidity, and temperature. Annual and seasonal NAO has been shown to influence seabird adult survival [30, 31]; breeding phenology [32]; reproductive success and breeding propensity [33]; foraging behaviour [33, 34]; and chick growth rates [34]. In the northwest Atlantic, positive NAO is associated with strong northwest winds, colder temperatures, less precipitation, higher ice cover, and increased storm activity [35–37]. Climate indices, like the NAO, can be useful in understanding effects of weather on wildlife because they provide a simplified measure of complex spatial and temporal variability in prevailing weather conditions within a region [38].

**Fig. 1:**
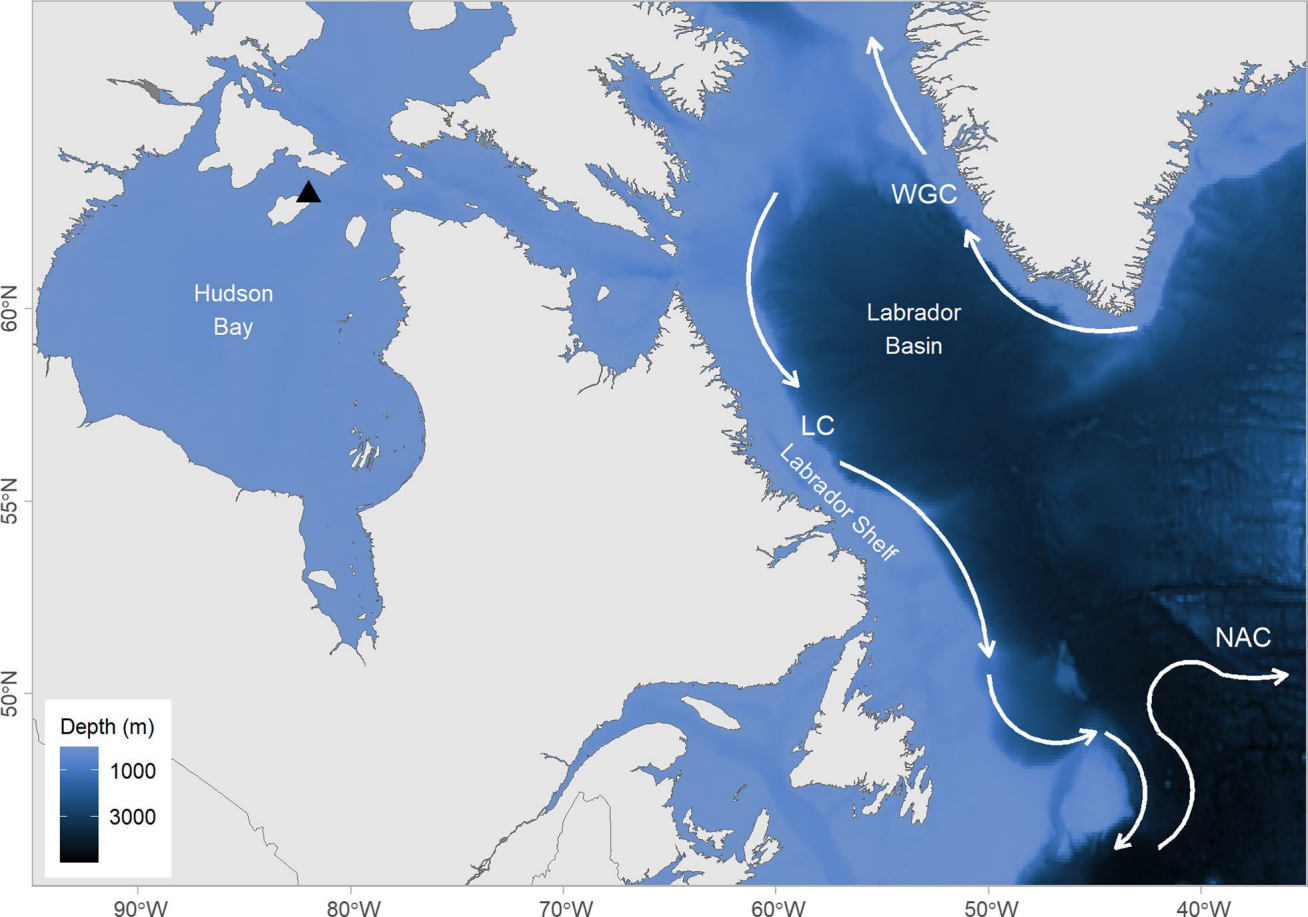
Study area map showing location of the breeding colony at Coats Island (black triangle) in Hudson Bay, the Labrador Shelf, and the Labrador Basin. White arrows indicate the flow of the Labrador Current (LC), the West Greenland Current (WGC), and North Atlantic Current, adapted from [44]

We used miniature temperature-depth loggers to study thick-billed murres originating from a breeding colony on Coats Island, in northern Hudson Bay (Fig. 1), which winter in the northwest Atlantic Ocean [39–41]. This globally-significant wintering area is shared by common (*Uria aalge*) and thick-billed murres, black-legged kittiwakes (*Rissa tridactyla*), Atlantic puffins (*Fratercula arctica*), and dovekies (*Alle alle*) breeding at colonies throughout Arctic and Atlantic Canada, as well as Greenland, Iceland, and Spitsbergen [8, 42, 43]. This region is dominated by cold water currents flowing along the Labrador and West Greenland Shelves, relatively warm and deep water of the Labrador Basin in between, and the warmer North Atlantic Current (Gulf Stream) to the south (Fig. 1).

We examined how marine habitat type (defined by sea surface temperature) and environmental conditions (moon illumination and climate conditions) influence behaviour and energetics of thick-billed murres during winter. We used measurements of sea surface temperature from leg-mounted temperature-depth-light recorders to classify individuals to three broad thermal habitats, which correspond well with the major ocean systems within the winter range of this population. We then examined how habitat class, day of year (DOY), moon illumination, and climate conditions (NAO) influenced winter strategies. Specifically, we looked for differences in daily activity budgets, daily energy expenditure, diving behaviour, and apparent energy intake, to determine how murres cope with the demands of winter within the marine habitats in their range.

## Methods

### Study species

Thick-billed murres are a long-lived, circumpolar seabird, which is considered an important indicator of Arctic marine ecosystems [24]. Murres undertake a short and highly seasonal breeding period (Jun-Aug), followed by a non-breeding period at sea (Sep-May) in Arctic or sub-Arctic regions. Winter tracking was initiated by deploying biologgers on thick-billed murres during breeding at Coats Island, Hudson Bay, Nunavut, Canada (62.95°N, 82.01°W), 2017 (n = 48) and 2018 (n = 36). This study focused on data recorded during January to March in 2018 and 2019, which coincides with the period when all individuals are present within their wintering range [41]. The datasets generated and analyzed during the current study are available in the Movebank Data Repository, https://doi.org/10.5441/001/1.81bs0nf7 [45].

### Temperature-depth-light recorders

In both years, we deployed LAT2800S temperature-depth-light recorders (Lotek, Newmarket, ON; 36-mm × 11-mm × 7.2-mm, 5.5-g). Loggers were programmed to collect light level, temperature, depth, and wet/dry state at 10-s intervals. All loggers were deployed on leg-bands attached to breeding adults, captured using a noose pole, while attending an egg or chick. Loggers were retrieved and downloaded during subsequent (one or two) breeding seasons.

### Estimating dive behaviour, SST, daily activity rates, and energetics

Dives were defined as any period when depth was at least 5 m. We calculated total time diving during four light categories according to the estimated solar angle at the mean position (average of dawn and dusk location estimates) for each individual on each day. Location estimates were obtained using the ‘probGLS’ package in R [46], with details provided in the supplementary material (Additional file 1: Table S1). Solar elevations were obtained using the ‘suncalc’ package [47] and classified as day (> 0°), civil twilight (0° to −6°), nautical twilight (−6° to −12°), and night (< −12°).

We estimated daily bird-detected sea surface temperature (SST) as the mean temperature measured for all at-surface data points during each day. To characterize at-surface points, we initially calculated the water temperature range experienced each day based on the 5th to 95th temperature measurement quantiles recorded during dives; this range was used to set upper and lower limits on potential SST. Murres were assumed to be at the surface of the water when: the temperature range experienced during every 180 s period was less than 0.5 °C; the bird was not diving; the tag was wet; and the temperature was within the water temperature range recorded by the tag that day (± 1 °C).

Murres were considered to be flying if the logger was dry for at least 60-s and the maximum temperature while dry was less than 7 °C. Murres were considered resting, with the tagged leg tucked in back feathers, if the maximum temperature was greater than 7 °C. This likely underestimates total time resting, because murres alternate which leg is tucked while resting [48]. We only treated identifiable leg-tucking events as resting to avoid making assumptions beyond what was detectable from the biologger. Daily activity rates were calculated for time flying, time diving, and time resting with the tagged leg tucked. All remaining time was classified as swimming for use in calculating daily energy expenditure (DEE).

We calculated DEE based on daily activity budgets, dive durations, and SST, using the equation from (Burke and Montevecchi [49], based on Elliott and Gaston [50]):

$$DEE = 508{*}T_{f} + 3.64{*}\sum \left( {1 - \frac{d}{{e^{1.23} }}} \right) + { }\left( {113 - 2.75{*}SST{ }} \right){* }T_{s} + { }\left( {72.2 - 2.75{*}SST} \right){*}T_{r}$$

where, *T*_*f*_ is time spent flying per day in hours, *d* is duration of each dive in minutes, *SST* is sea surface temperature in °C, *T*_*s*_ is time spent actively swimming in hours, and *T*_*r*_ is time spent resting on the water in hours. Note that the coefficient for energy expended as a function of dive duration (3.64 kJ) differs from Elliott and Gaston [50], in which the published coefficient was not correctly converted from watts to kilojoules from Elliott et al. [19].

We calculated an apparent energy intake rate assuming that murres are balancing their energy budget over a 5-day interval. The apparent energy intake (AEI) rate was based on the 5-day moving averages of DEE and time spent diving:

$$AEI = { }\frac{{DEE_{5} * 1/E}}{{Td_{5} }}$$

where, DEE_5_ is the 5-day rolling average of daily energy expenditure, Td_5_ is the 5-day rolling average of time spent diving. E is a constant to correct for assimilation efficiency (73%) of prey items [51].

### Classification of habitat states and habitat distribution

Three thermal habitat states—Cold, Warm, and Warmer—were defined using a hidden Markov model (HMM), with mean daily SST as a predictor variable. A three-state model was the minimum number of states required to obtain non-overlapping state distributions. State distributions were modelled using a normal distribution, with initial probability distributions (mean ± SD) of −1 ± 3 °C (Cold), 3 ± 3 °C (Warm), and 8 ± 3 °C (Warmer); varying these starting values by ± 1 °C had no affect on predicted state distributions. Daily transition probabilities among states were modelled as a function of four potential main effects: year (2018 or 2019), day of year, moon illumination, and NAO. We obtained moon illumination based on the fraction of the moon visible on each date, values ranged from 0 (new moon) to 1 (full moon), using the ‘suncalc’ package [47]. Daily NAO values were obtained from the United States National Weather Service Climate Prediction Center (https://www.cpc.ncep.noaa.gov/products/precip/CWlink/pna/nao.shtml), which uses the rotated principal component analysis procedure [52]. AIC was used to identify the most parsimonious model among all combinations of main effects on the transition probabilities. The HMM models were run using the ‘momentuHMM’ package, version 1.5.2 [53]. The Viterbi algorithm was used to assign each data point to one of the three habitat states [53]. Model checking was performed by simulating observations from the fitted model and comparing observed data to the expected quantiles and autocorrelation function from the simulated data [54].

We used monthly remotely-sensed SST measurements to estimate the spatial distribution of each habitat class within the study area. We extracted environmental data from a 0.25° × 0.25° grid within the study area from Copernicus Marine Service Global Ocean Physics Reanalysis (https://resources.marine.copernicus.eu/product-detail/GLOBAL_MULTIYEAR_PHY_001_030). We calculated the probability density for each state based on the SST value of each monthly raster cell and assigned each cell to the state with the highest probability.

### Statistical analysis

We examined dive depth profiles across habitat class and light category. We calculated the percentage of all dives within each habitat that occurred within 10-m depth categories for each of the four light classes.

We used generalized linear mixed models (GLMM) to test for effects of habitat type, DOY, moon phase, and daily NAO on daily activity rates (flying and diving), the proportion of total time diving during different light conditions (day, civil twilight, nautical twilight, and night), and energetics (DEE and AEI). Preliminary data analysis showed no evidence of differences in diving, activity budgets, or energetics between males and females, therefore we did not include sex in the analysis. Models included all two-way interactions between habitat and other main effects (DOY, moon illumination, and NAO). Models of dive times and activity times were fit using a beta distribution with a logit link function. For daily activity rates, the response variables (flying and diving) were divided by 24 h to normalize values between 0 and 1. For proportion of time diving under different light conditions, all four response variables (daylight diving, civil twilight diving, nautical twilight diving, and night diving) were divided by the total time spent diving that day to normalize values between 0 and 1. Where the response variable contained more than 5% zero values (time flying, diving during nautical twilight, diving during night) we included zero inflation (ZI) parameters as well as conditional parameters. When less than 5% of the response values had zero values (time diving, diving during daylight, and diving during civil twilight) we added 1 min of time to response values to fit models without zero inflation. Models of energetics (DEE and AEI) were fit with a Gamma distribution, with a log link function, to ensure that parameter estimates were positive. Individual identity was included as a random effect in all models. An Ornstein–Uhlenbeck covariance structure was used to account for temporal autocorrelation within individuals.

GLMM models were fit using the ‘glmmTMB’ package in R [55]. Models were compared using Akaike Information Criterion (AIC), where multiple models had < 2 ∆AIC the model with the fewest parameters was selected as the most parsimonious model. For models that included ZI terms model selection was performed in two stages, first identifying the best supported model for only ZI component and then holding ZI terms constant to identify the best supported conditional terms. Parameter estimates in supplementary tables are presented on the link scale ± SE. Model predictions provided in the text are estimated marginal means with 95% confidence intervals on the response scale. GLMM model fits were evaluated using posterior predictive checks with the ‘performance’ package in R [56].

## Results

We recovered winter biologging data from 34 murres in 2018 and 20 murres in 2019, including 10 birds that were tracked in both years. The majority of tracks covered the entire winter period (96%), except for two tracks that ended during March. Partial tracks were included in the analysis. Sample size of tracks was split relatively evenly between males (n = 25) and females (n = 29).

### Habitat classification and physical oceanography

**Fig. 2:**
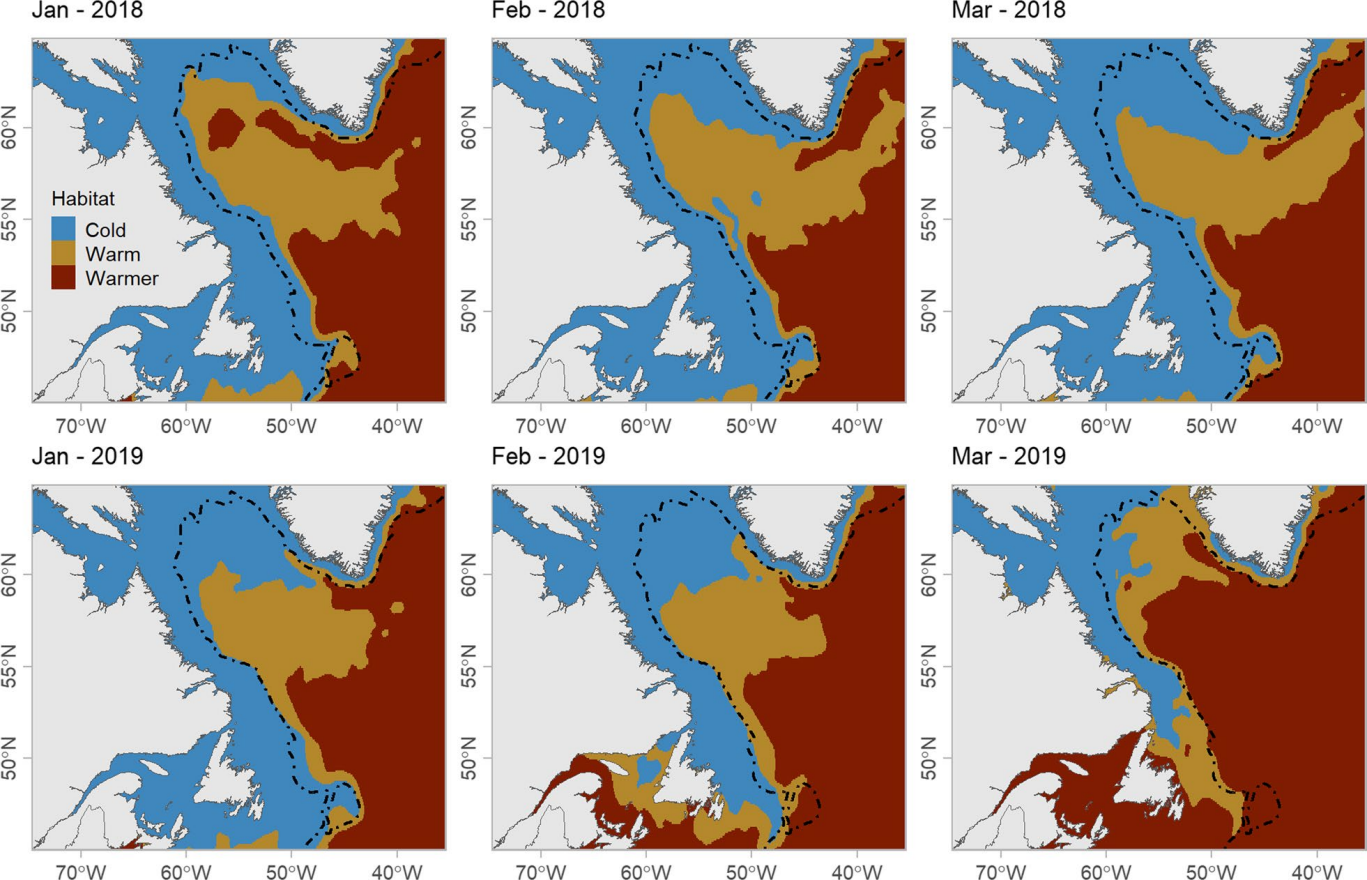
Estimated monthly spatial distribution of the three habitat types—Cold water, Warm water, and Warmer water—identified using a hidden Markov model, for winter 2018 and 2019. Dashed line indicates the 1000 m shelf break

Mean SST within the three habitat states from the HMM was −0.1 °C (± 1.2 °C) for Cold water, 3.1 °C (± 0.3 °C) for Warm water, and 6.5 °C (± 2.4 °C) for Warmer water. The spatial distribution of these habitat states broadly corresponded to the major currents within the Northwest Atlantic (Fig. 2). The Cold water habitat was primarily located in shelf regions along the Labrador, Newfoundland and Greenland coasts; this habitat corresponds to cold-water carried by the Labrador, West Greenland, and East Greenland Currents (Fig. 1) [57]. The Warm habitat was located in the deep water of the Labrador Basin. The Warmer water habitat occurred primarily in the south and east extent of the winter range, and includes water from the North Atlantic Current [57]. This distribution of habitat was relatively consistent throughout the study period, except for Mar 2019, when the warmer water habitat intruded farther into the Labrador Basin (Fig. 2). A detailed comparison of physical oceanographic features associated with the habitat classes is provided in the supplementary material (Additional file 1: Fig S1).

### Murre habitat use and transition probabilities

**Fig. 3:**
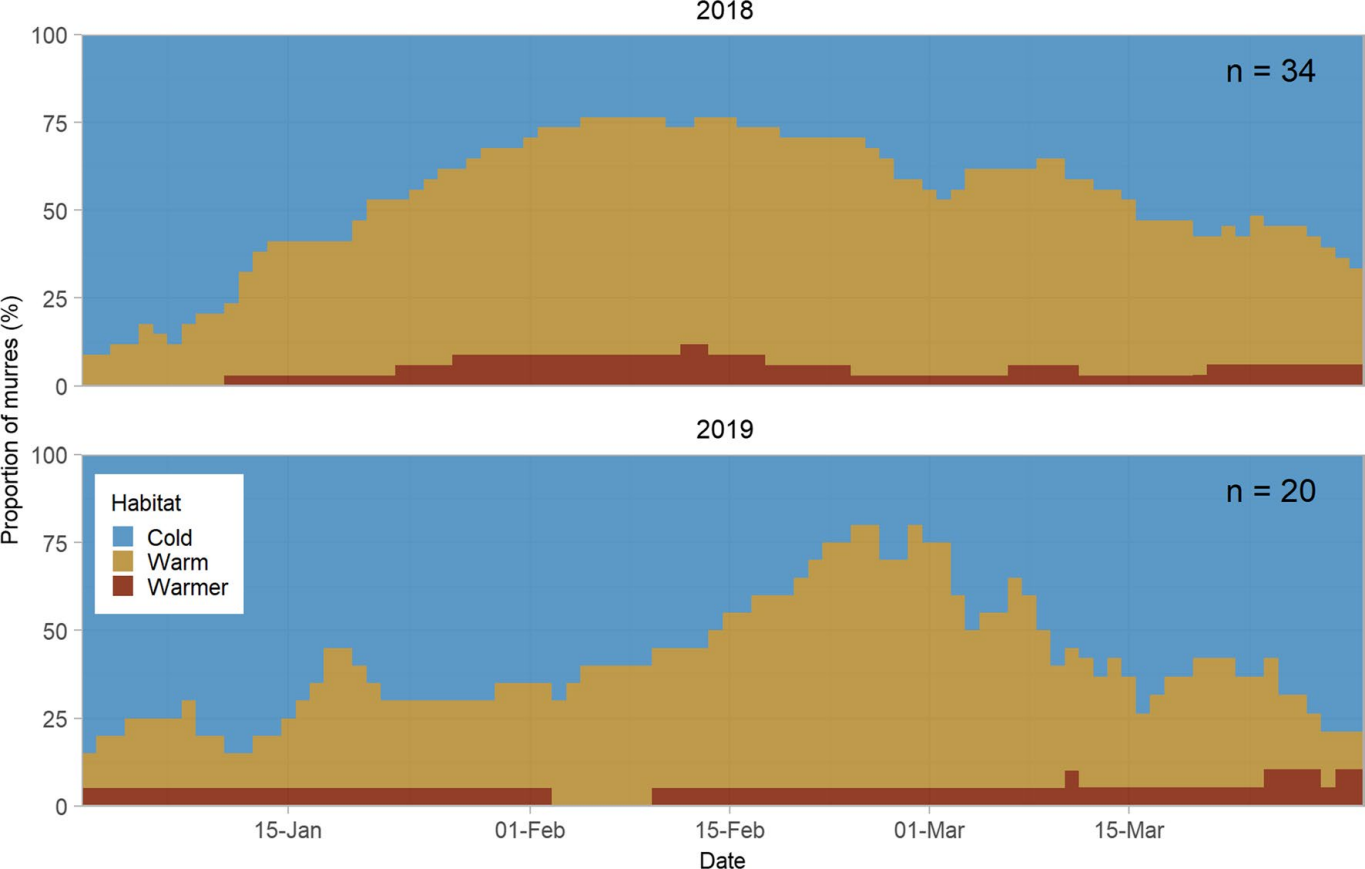
The proportion of thick-billed murres tracked from Coats Island, NU, Canada, using each habitat type by date and year in winter (Jan–Mar) of 2018 and 2019. Habitat types were determined using a hidden Markov model with sea surface temperature measured using leg mounted temperature-depth-light recorders, as the observed data

In both years, the proportion of murres using Cold water was highest in January and March, while use of Warm water peaked in February (Fig. 3). On average, murres spent 46 days (range = 5–90) in Cold water, with six individuals (11%) staying in Cold water through the entire winter period (Jan 1–Mar 31). The mean time spent in Warm water was 39 days (range = 0–85) and 87% of tracked murres spent some time in Warm water. Use of Warmer water was low throughout the winter; only 17% of murres spent any time in Warmer water, with mean time of only 5 days (range = 0–64, Additional file 1: Fig S2). Cold and Warm water habitat were used at similar rates and the majority of tracked murres, 89%, switched between at least two habitats during winter (Additional file 1: Fig S2). For the 10 murres tracked for two years, one individual used Cold water exclusively in both years and the other nine individuals used a mix of habitats in both years. However, the relative proportion of time spent in Cold and Warm habitat differed between years for five individuals (Fisher’s exact tests, *p* < 0.05). Only one of the multi-year birds used Warmer water, and this individual used Warmer water in both years.

Daily transition probabilities were highest between Cold and Warm water (Cold-Warm 0.033, Warm-Cold 0.024). Daily transitions from either Cold or Warm water to Warmer water had a relatively low probability (Cold-Warmer 0.001, Warm-Warmer 0.003). Transitions probabilities were higher from Warm to Cold (0.84, CI = 0.36–1.32) in 2019. Murres in Warm water were more likely to transition to Cold water when NAO was negative (−0.63, CI = −0.96–0.30) and when moon illumination was lower (−0.73, CI = −1.40–0.06).

Because murres in our study made minimal use of the Warmer water habitat (Additional file 1: Fig S2), only the Cold and Warm water habitats were considered in the remaining analysis.

### Daily activity rates

**Fig. 4:**
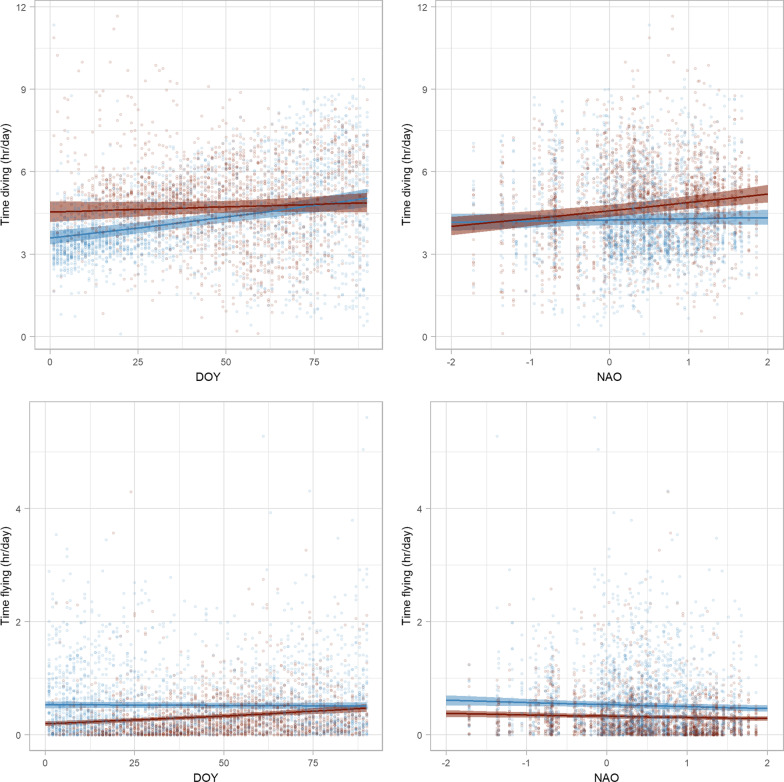
Predicted marginal effects of day of year (DOY) and the North Atlantic Oscillation (NAO) on time spent flying and diving for thick-billed murres in Cold water (blue) and Warm water (red) habitat in the Northwest Atlantic. Solid lines are predicted marginal means from a generalized linear mixed effects model, shaded areas are 95% confidence intervals, and points are observed daily observations. Note that y-axis ranges differ among rows

Murres spent an average of 3–6 h diving per day throughout winter (Additional file 1: Fig S3). DOY and NAO influenced total time diving per day, but these effects differed between habitats (Fig. 4, Additional file 1: Tables S2, S3). In Cold water, murres increased the amount of time diving from 3.6 h/day (CI = 3.4–3.9 h/day) in early winter to 5.0 h/day (CI = 4.5–5.2 h/day) in late winter. This increase in time diving with DOY is likely driven by increased availability of daylight later in winter. In Warm water, there was no effect of DOY on time diving; however, NAO had a strong positive effect on total time diving. At NAO −2 murres in Warm water were expected to spend 4.0 h/day (CI = 3.7–4.4 h/day) diving, at NAO + 2 murres were expected to spend 5.2 h/day (CI = 4.9–5.5 h/day) diving. This could indicate that murres in Warm water were able to forage more efficiently when NAO was negative, when a higher proportion of total dive time occurred at night.

Murres spent less than 1 h flying per day throughout winter (Additional file 1: Fig S3). Time spent flying differed between the two habitats, and there were interactions between habitat and DOY and habitat and NAO (Fig. 4, Additional file 1: Tables S2, S3). Murres spent more time flying (0.52 h/day, CI = 0.47–0.57) in Cold water than in Warm water (0.32 h/day, CI = 0.29–0.35). In Warm water, murres doubled the amount of time flying per day from 0.20 h/day (CI = 0.16–0.24 h/day) in early winter to 0.48 h/day (CI = 0.41–0.55 h/day) in late winter; while DOY had no affect on time flying in Cold water (Fig. 4). In both habitats, murres flew less when NAO was more positive. Increased wind associated with positive NAO phases could limit flying by murres throughout their winter range.

### Proportion of time diving and dive depths under different light conditions

**Fig. 5:**
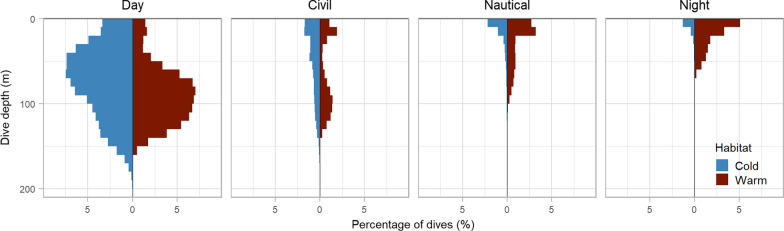
Distribution of dive depths by habitat type (Cold–blue, Warm–red) and light level. Each panel shows the percentage of dives at 10 m depth intervals, values across panels sum to 100 for each habitat type

The majority of dives occurred during the day in both habitats (Fig. 5); however, the proportion of dives during the day was much higher in Cold water, 81%, than in Warm water, 62%. Murres in Warm water made a higher proportion of their dives during nautical twilight, 12%, and at night, 14%. Day dives were primarily concentrated between 40 and 100 m deep in Cold water, while murres in Warm water made deeper dives during the day (70–130 m). Dive depths attenuated with light availability in both habitats; most dives during nautical twilight and at night were less than 20 m deep.

**Fig. 6:**
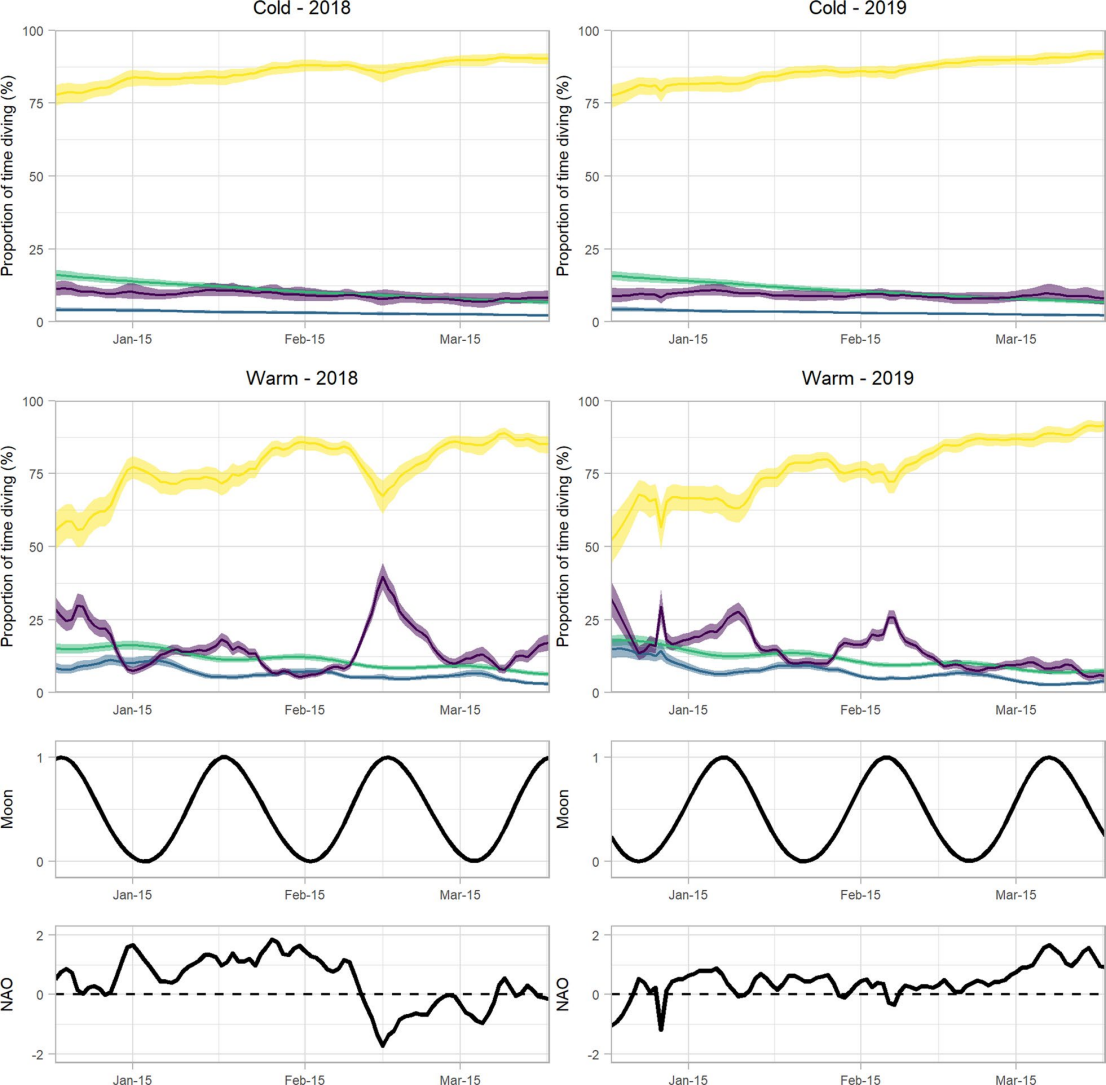
Predicted proportion of time diving (%) for thick-billed murres in winter, based on time of day (yellow = daylight, green = civil twilight, light blue = nautical twilight, dark blue = night), day of the year (DOY), habitat type (Cold or Warm), moon illumination (0 = new, 1 = full) and North Atlantic Oscillation (NAO) index. In the two upper plots, the solid line shows the mean prediction and shaded areas are the 95% confidence interval

**Fig. 7:**
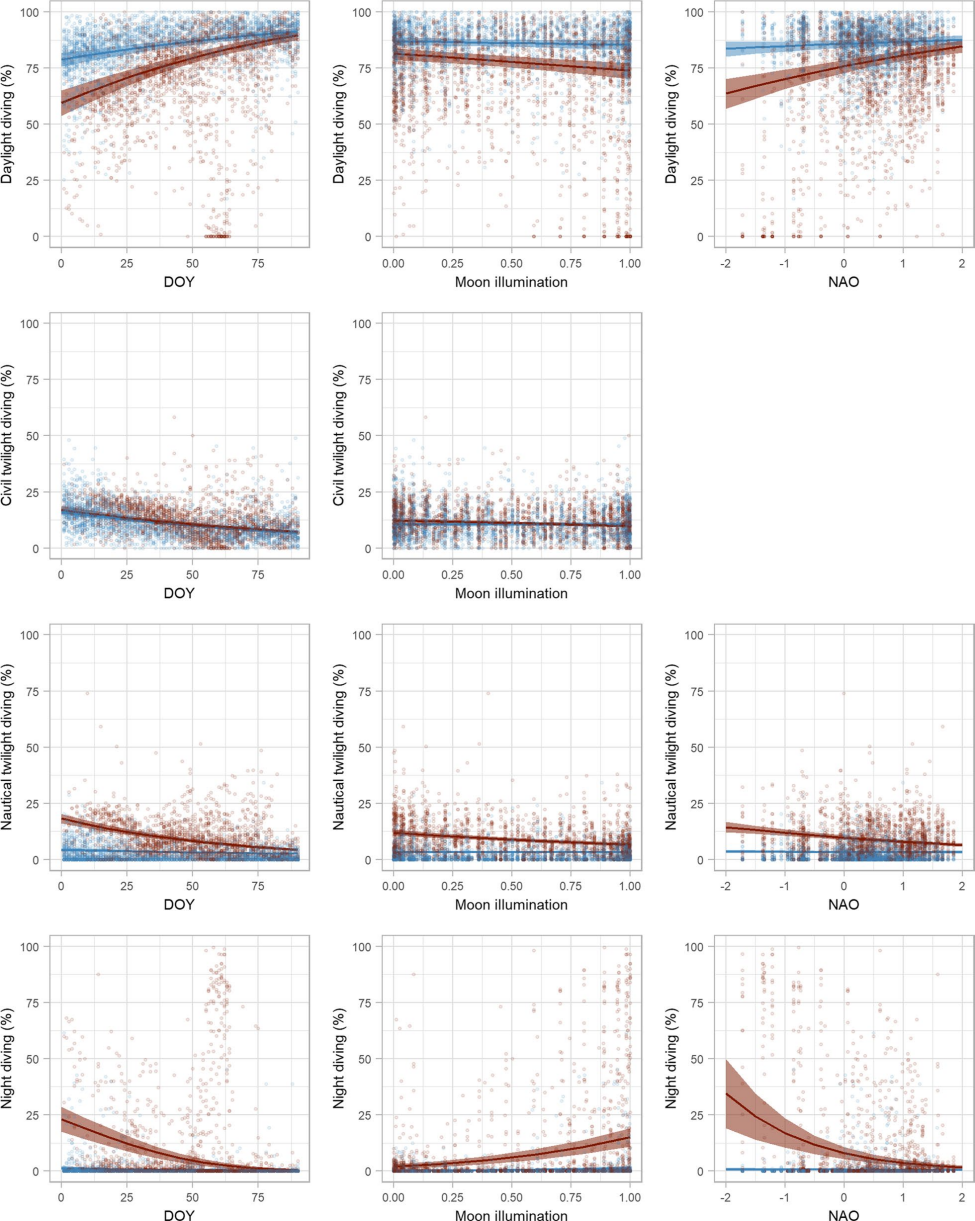
Predicted marginal effects of day of year (DOY), moon illumination (0: new moon, 1: full moon), and the North Atlantic Oscillation (NAO) on proportion of time diving (%) during daylight, civil twilight, nautical twilight, and night for thick-billed murres in Cold water (blue) and Warm water (red) habitat in the Northwest Atlantic. Solid lines are predicted marginal means from a generalized linear mixed effects model, shaded areas are 95% confidence intervals, and points are daily observations

Habitat type and DOY influenced proportion of time diving under all light conditions (Additional file 1: Tables S4, S5). Most diving occurred during daylight for murres in both habitats (Fig. 6); however, daylight diving was more prevalent for murres in Cold water (Fig. 6). The proportion of diving that occurred during daylight increased with DOY in both habitats, but the rate of increase was greater in Warm water than in Cold water (Fig. 7). Diving under low light conditions (civil twilight, nautical twilight, and night) was more prevalent for murres in Warm water, where murres made a significantly higher proportion of dives under low light conditions in early winter. In both habitats the proportion of diving during civil and nautical twilight declined with DOY, and this decline for nautical twilight diving was greater in Warm water. Night diving declined with DOY in Warm water, but not in Cold water. Short day lengths in early winter apparently limit day light diving in both habitats, but murres in Warm water compensated by increasing the proportion of time diving under low light conditions.

Increased moon illumination and negative NAO both contributed to an increased proportion of time diving under low light conditions (nautical twilight and night) by murres in Warm water, but had no effect on diving behaviour of murres in Cold water (Fig. 7, Additional file 1: Tables S4, S5). When the moon was more illuminated, murres in Warm water increased the proportion of their diving at night and decreased the proportion of diving that occurred during other light conditions. In Warm water, NAO had a strong positive effect on proportion of diving that occurred during the day and a strong negative effect on time diving during nautical twilight and at night. The marginal effect of NAO on timing of diving for murres in Warm water was remarkable: under NAO + 2, the vast majority of diving occurred during the day (85%, CI = 82–87%) and night diving was minimal (2%, 1–3%), while under NAO −2, the proportion of time diving at night increased to 34% (18–50%) the predicted proportion of diving during the day declined to 64% (57–70%).

### Energetics

**Fig. 8:**
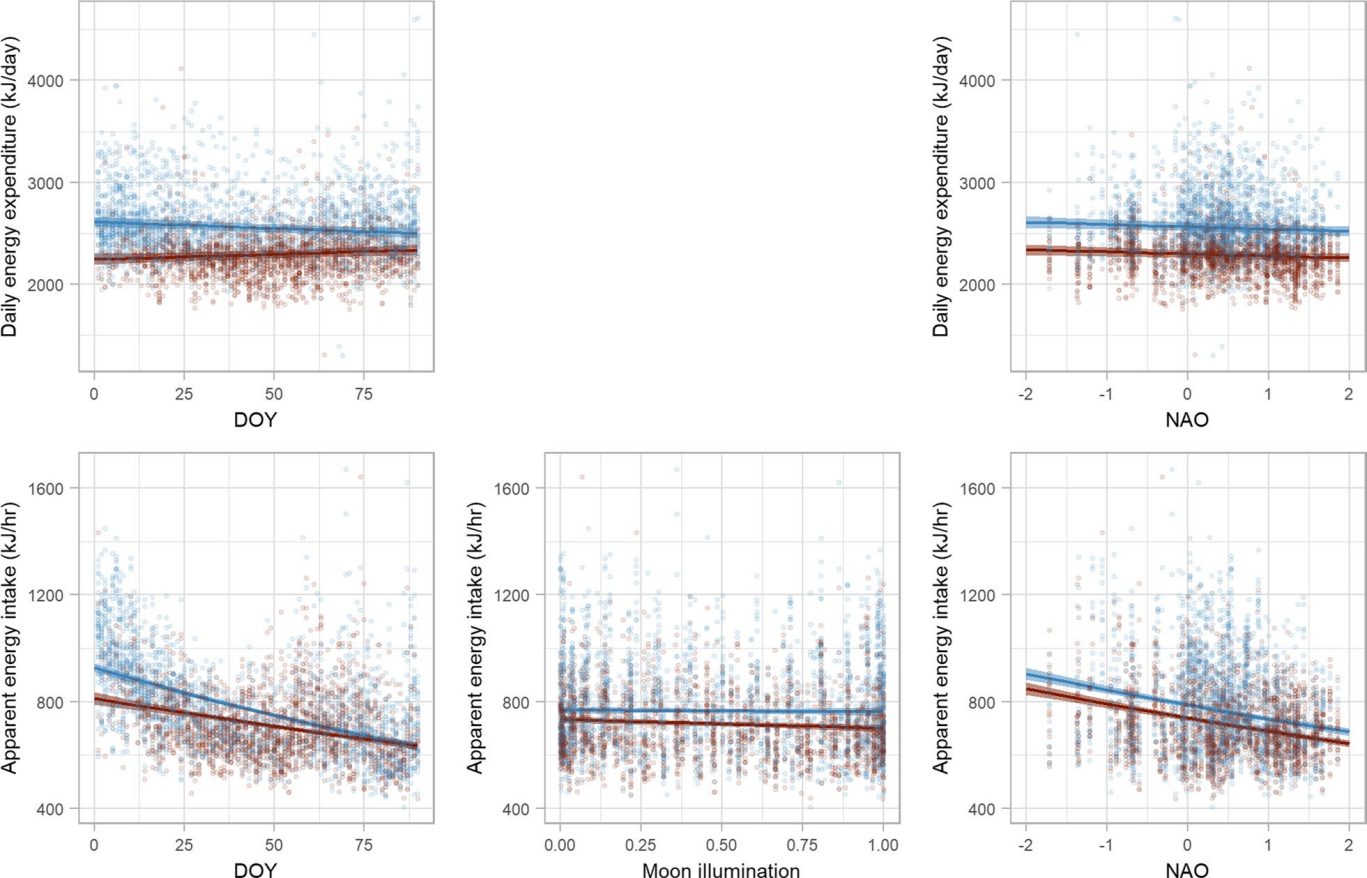
Predicted marginal effects of day of year (DOY), moon illumination (0: new moon, 1: full moon), and the North Atlantic Oscillation (NAO) on daily energy expenditure (DEE) and apparent energy expenditure (AEI) for thick-billed murres in Cold water (blue) and Warm water (red) habitat in the Northwest Atlantic. Solid lines are predicted marginal means from a generalized linear mixed effects model, shaded areas are 95% confidence intervals, and points are observed daily observations. Note that y-axis ranges change among rows

Murres had higher DEE in Cold water, 2558 kJ/day (CI = 2517–2598) than in Warm water, 2292 kJ/day (CI = 2254–2330, Additional file 1: Fig S4, Tables S6, S7). This difference was driven by a combination of additional time flying and the lower SST in the Cold water habitat. DEE declined during the winter in Cold water and increased slightly in Warm water (Fig. 8). NAO had a negative effect on DEE for murres in both habitats, which can be attributed to the decline in time flying under positive NAO conditions.

Apparent energy intake was higher in Cold water, 765 kJ/h diving (CI = 756–773) than in Warm water, 717 kJ/h diving (CI = 708–726, Additional file 1: Fig S4, Tables S6, S7). AEI declined with DOY in both habitats; however, the rate of decline was greater in Cold water than Warm water (Fig. 8). By the end of winter, AEI was similar in both habitats. Moon illumination had a modest negative effect on AEI in Warm water, but there was no moon effect in Cold water. NAO had a strong negative affect on AEI in both habitats; when NAO was positive, murres in both habitats had lower foraging efficiency, and spent relatively more time diving to meet their estimated DEE.

## Discussion

Marine habitat had strong effects on dive behaviour, daily activity rates, and energetics. Murres regularly used two habitat types during winter, Cold water that occurs along the Labrador and Greenland shelves and Warm water within the Labrador Basin. Murres had significant among and within-individual flexibility to exploit these two habitats, which apparently support different foraging strategies for surviving the high energetic costs of winter. Wintering in the Cold shelf water had higher energetic costs associated with thermoregulation and increased flight activity; however, these costs were evidently offset by higher apparent energy intake rates during early winter, requiring less diving each day. The Warm basin water had lower energetic costs, but required murres to spend longer foraging each day, including foraging under low light conditions during nautical twilight and at night.

Murres from our study population occasionally used Warmer water within the North Atlantic current; however, the proportion of tracked murres using this habitat was relatively small, precluding a detailed analysis of behaviour and energetics within this habitat. While murres in our population made relatively little use of this Warmer water in mid-North Atlantic, this region does support wintering murres from other colonies [42] and is a persistent year-round hotspot for numerous seabird species [58].

Murres in Cold water apparently used a high-energy foraging strategy. Murres spent the most time flying and the least time diving while in this habitat. Additional flight time and costs of thermoregulation both contributed to higher DEE in Cold water. Increased flying by murres in this habitat is likely an investment in more energy searching for high quality prey patches. In spite of these increased energetic costs, in Cold water, murres spent less time diving through most of the winter than those in Warm water, implying a higher apparent energy intake rate and higher foraging efficiency to maintain their energy balance. This high-energy strategy could be susceptible to sudden declines in prey availability, and be especially risky when extreme weather or ice conditions prohibit travelling to areas with better prey conditions. Coastal ice build-up and sustained northeasterly winds have contributed to wrecks of thick-billed murres in Newfoundland, where murres trapped in coastal bays by ice starved within 2–3 days [43]. Similarly, many seabirds wintering in the North Atlantic are vulnerable to extended periods of stormy weather, where high wind and rough seas are thought to limit birds ability to forage or access prey over extended periods of time [8, 11, 59]. Body temperature increases during flight [60], particularly for aquatic species with high wing loading [61]. In Cold water habitat, increased flying may have additional benefits for thermoregulation as well as locating prey.

In contrast, murres in Warm water used a low-energy strategy, expending less energy on flight and thermoregulation in favour of more time spent diving for prey. For murres, decreasing time searching for prey in the air and rather investing more time searching underwater would have significant energetic savings as the costs of diving are comparable to swimming at the surface [19]. Passive foraging should only be a more profitable at low prey density [17, 20, 21]. If prey density is low, and murres are foraging passively to conserve energy, then Warm water habitat has the additional benefit of reduced energy expenditure on thermoregulation. In both years, use of Warm water peaked in mid-winter (February), potentially because murres move out of Cold water as accessible prey density declines through winter (either due to increased ice cover or because prey move to depths greater than 200 m) and return to Cold water in March as the time available for foraging during daylight increases.

Murres only dove significantly at night in Warm water. This difference could be because in Cold water they were able to meet their daily energy needs during daylight while murres in Warm water could not. Alternatively, the relatively shallow Cold water habitat may not support significant densities of DVM prey. Murres in Warm water increased their night diving effort in response to increased moon illumination (full moon) and weak NAO conditions. We propose that this was a response to increased opportunity to benefit from DVM when light conditions were favourable for diving at night. Interestingly, murres in Warm water reduced diving during nautical twilight when moon illumination was high but increased it when NAO was positive. Potentially, murres may maximize diving under the fading light conditions of nautical twilight when there is no moonlight but clear skies, but wait for full night conditions when the moon is brighter, or the arrival of DVM prey in shallow water is delayed under high moon illumination. Limited night foraging in Cold habitats could have important climate change implications. If Cold shelf habitat does not support foraging at night, then individuals in this habitat cannot switch to nocturnal foraging as northward range shifts decrease time available for foraging during the day, creating a habitat-mediated photic barrier to range shifts [62].

The rapid response of murres to daily changes in NAO could be an indication that the effect of NAO on murre behaviour is mediated through weather, specifically wind or cloud cover, which could affect both the energetic costs of flight and the foraging ability of murres. Other indirect mechanisms of NAO forcing through effects on the food web would likely occur over a longer time scale than what was tested here. DEE and AEI were both higher under negative NAO conditions; we attribute this relationship to increased time flying during weak NAO. Wind speeds in the Northwest Atlantic are higher under positive NAO conditions, in response murres likely reduce time flying due to increased wind [25, 63]. This could simultaneously reduce DEE and AEI if murres are limited in their ability to search out prey. Probably the most dramatic results associated with NAO were the marked increase in nocturnal foraging, and concurrent decline in total time foraging, in Warm water when NAO was negative. We interpret this as increased foraging efficiency at night. Negative NAO is associated with decreased wind in the Northwest Atlantic. This could promote nocturnal foraging if decreased wind allows stratification of surface water layers [27], promoting increased plankton growth and greater biomass of DVM. Less wind could also cause reduced turbulence and a thinner active mixing layer at the surface [64], forcing prey to migrate closer to the surface at night where they would be more accessible to diving murres [65]. Given that our time series of murre behaviour only covers two winters, during which strongly negative NAO conditions occurred infrequently (primarily late-Feb to late-Mar 2018), it is possible that the effect of NAO observed here arose from a single anomalous event that may not re-occur with additional monitoring.

Given the differences in oceanography and foraging behaviour between the Cold (shelf) and Warm (basin) habitats observed in our study, it is likely that murres target different prey within these habitats. Relatively little is known about the winter diet of thick-billed murres, and what sampling has occurred is biased towards murres collected by hunters in coastal waters around Newfoundland and western Greenland [66–68]. Early studies of winter diet reported a shift from predominantly fish and squid in early winter to amphipods and euphausiids in Jan–Mar [66]. Invertebrate prey include amphipods (*Parathemisto* spp.), euphasiids (*Thysanoessa* spp.), squid *(Gonatus fabricii*) and polychaetes (*Nereis pelagica*), while fish species identified in winter diets include Arctic cod (*Boreogadus saida*), Atlantic cod (*Gadus morhua*), capelin (*Mallotus villosus*), and Northern sandlance (*Ammodytes dubius*) [66–69]. Isotopic analysis indicated that thick-billed murres collected around Newfoundland fed on a mix of fish and invertebrate prey in winter, with a higher proportion of invertebrates in their diet than during the breeding season [69]. Murres collected near Nuuk, Greenland, switched from a diet dominated by fish, primarily capelin, in October to one dominated by crustaceans in March [67], similar to trends reported in Newfoundland. We found an increase in time spent diving and a decrease in AEI for murres in Cold water through winter, which would be consistent with a switch from fish to crustaceans as observed in these other studies. Myctophids (*Benthosema glaciale*) are abundant and evenly distributed within the Labrador Sea [70–72], residing in the bathypelagic zone during the day and migrating into the epipelagic layer at night. Myctophids are an important prey species for many seabirds that forage nocturnally [73] and could be an important component of the diet of murres in the Labrador Sea.

Our results for thick-billed murres show similarities with other studies. The distribution of thick-billed murres using Cold water in our study overlapped with the winter distribution of common murres on the Grand Banks [49]. Both species spent similar time flying (COMU: 0.5 h/day, TBMU: 0.6 h/day), but thick-billed murres (Cold: 4 h/day, Warm: 4 h/day) spent more time diving than common murres (3 h/day). Similar to thick-billed murres in Cold water in our study, common murres on the Grand Banks spent minimal time diving at night (3% of total dive time in Dec-Feb). Common murres wintering in the North Sea, reside in warmer (6–8 °C) water than the Cold and Warm habitats used by thick-billed murres in our study [74]. In the North Sea, common murres spent very little time flying (0.2 h/day), a significant amount of time diving (4–5 h/day), and also engaged in significant night diving (1.2 h/day) from Dec to Feb [74], similar to murres using the Warm Labrador Basin water in our study. These similarities suggest that a high-energy strategy in cold water and a low-energy strategy in warm water may be consistent among murres from different populations and in different wintering areas.

## Conclusions

The availability of different marine habitats in the Northwest Atlantic may be advantageous to mobile marine predators, like thick-billed murres, by allowing them to match their winter habitat use to individual condition and changes in local environment. Whereas many species are only able to choose a single strategy to cope with the polar winter, murres in this population are able to switch habitats and adapt their foraging behaviour to that habitat. Dispersal across thermal gradients in response to individual variation in thermal preference has been linked to phenotype dependent survival rates in lizards [75]. Assuming individuals within this population are exhibiting habitat matching, there are potentially three distinct winter phenotypes: warm-water specialists, cold-water specialists, and habitat generalists [76, 77]. Additional multi-year tracking would help to determine intrinsic and extrinsic drivers of habitat use during winter. The next step is to understand the fitness consequences of these phenotypes, and how those fitness consequences could change with anticipated marine climate change in the coming century.

## Supplementary Information

**Additional file 1**. Supplementary tables and figures.

## Data Availability

The datasets generated and/or analyzed during the current study are available in the Movebank Data Repository, https://doi.org/10.5441/001/1.81bs0nf7
