# Supplementary material for: Behavioural flexibility in an Arctic seabird using two distinct marine habitats to survive the energetic constraints of winter
Source: Mov Ecol. 2022 Nov 3;10:45. doi: 10.1186/s40462-022-00344-3 (PMC9635182; doi:10.1186/s40462-022-00344-3)
Supplement: Supplementary file 1 — Additional file 1. Supplementary tables and figures. [file 40462_2022_344_MOESM1_ESM.docx]

# Supplementary Material

## Geolocator estimates used to determine solar angle

For location estimates, we summarized maximum-recorded light levels at 5-minute intervals prior to estimating twilights. Twilight times were estimated using the threshold method in the ‘TwGeos’ package [1]. We defined two behavioural modes, flying and on water. Flying was defined as any period where the sensor was dry and tag temperature was less than 5°C; this temperature threshold was used to prevent periods of leg-tucking from being falsely classified as flying [2]. Location estimates were calculated using a probabilistic algorithm that incorporates data from twilight estimates, a land-sea mask, sea surface temperature, and movement rates in order to generate more realistic estimates of seabird positions from geolocators [3,4]. Location estimates were constrained to be over water with less than 90% ice cover within each 0.25° x 0.25° raster cell. We also included sea surface temperature (NOAA High Resolution SST NOAA/OAR/ESRL PSL, https://psl.noaa.gov/, Reynolds et al. 2007) matching in location estimates. Full details of the parameters used in the probabilistic algorithm are provided Table S1.

Table S1. Parameter values used in the probGLS model to estimate locations from temperature-depth-light loggers.

| Parameter | Value |
| --- | --- |
| Light threshold^1^ | 175 |
| Solar angle range (°) | -6 to -1 |
| Speed – In flight (m/s) |  |
| Mean | 15 |
| SD | 5 |
| Max | 25 |
| Speed – On water (m/s) |  |
| Mean | 1 |
| SD | 1.3 |
| Max | 5 |
| SST SD (°C) | 0.1 |
| Max SST difference (°C) | 2 |
| Spring equinox (days) | -21 to 14 |
| Fall equinox (days) | -14 to 21 |
| Ice concentration cut-off (%) | 90 |
| Boundary box (°) | -110W, -10W, 30N, 75N |

^1^ Unitless values specific to tag model.

## Physical oceanographic characteristics of the habitat classes

We used bathymetry and oceanographic models to examine the spatial distribution and physical oceanography of the habitat classes defined from the HMM. We extracted environmental data from a 0.25⁰ x 0.25° grid within the study area (40°-70° N and 35-75°W). Bathymetry was obtained from ETOPO1 Global Relief Model (<https://www.ngdc.noaa.gov/mgg/global/>). Monthly SST, ice cover concentration, mixed layer depth (MLD), sea surface height (SSH), and current velocity were obtained from Copernicus Marine Service Global Ocean Physics Reanalysis (GLOBAL_ANALYSIS_FORECAST_PHY_001_030). We calculated the probability density function for each state based on the SST value of each monthly raster cell and assigned each cell to the state with the highest probability. We compared physical oceanography of the three habitat classes using density plots, medians, and inter-quartile ranges.

The three habitats identified from the HMM were also associated with differences in other physical oceanographic habitat characteristics within the Northwest Atlantic, in addition to SST (Figure S1). Cold water habitat was primarily located over shelf water (-233 m; IQR = -470 to -114 m), had a shallower mixed layer depth (33 m; IQR = 20 to 46 m), higher sea surface height (-0.53 m; IQR = -0.71 to -0.45 m), and more ice cover (22%; IQR = 0.4 to 77%) than the other two habitats. Warm water habitat was relatively deep (-3200 m; IQR = -3400 to -2700 m) and ice-free (0.0%; IQR = 0 – 0.2%), with a deeper mixed layer (216 m; IQR = 142 to 421 m) and lower sea surface height (-1.04 m; IQR = -1.1 to -0.99 m) than the Cold water habitat. The Warmer water habitat had the deepest bathymetry (-3450 m; IQR = -4200 to -2700 m), no ice cover (0.0%), intermediate mixed layer depth (145 m; IQR = 102 to 203) and intermediate sea surface height (-0.92 m; IQR = -0.99 to -0.67). Current velocities overlapped for the three habitat classes.


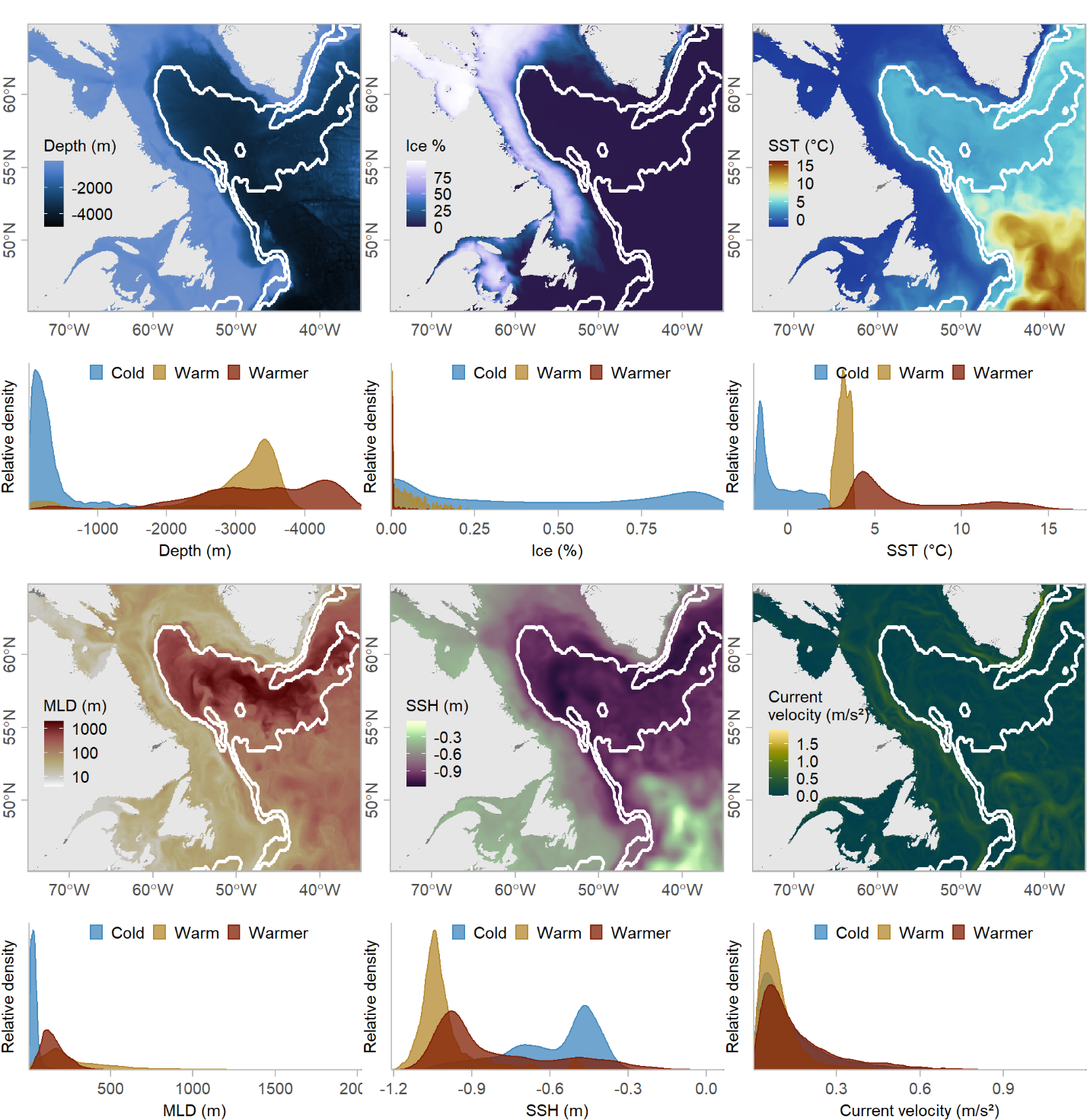
Figure S1. Maps showing physical oceanographic characteristics – sea surface temperature (SST), bathymetry, ice cover, mixed layer depth (MLD), sea surface height (SSH), and current velocity – within the study area for Feb 2018. Density plots show the distribution of each variable by habitat type at sample points throughout the study period (Jan-Mar, 2018 and Jan-Mar 2019). Oceanographic data from Copernicus Marine Service, GLOBAL_REANALYSIS_PHY_001_030. The white outlines show the state boundaries for Feb 2018 as shown in Figure 2.


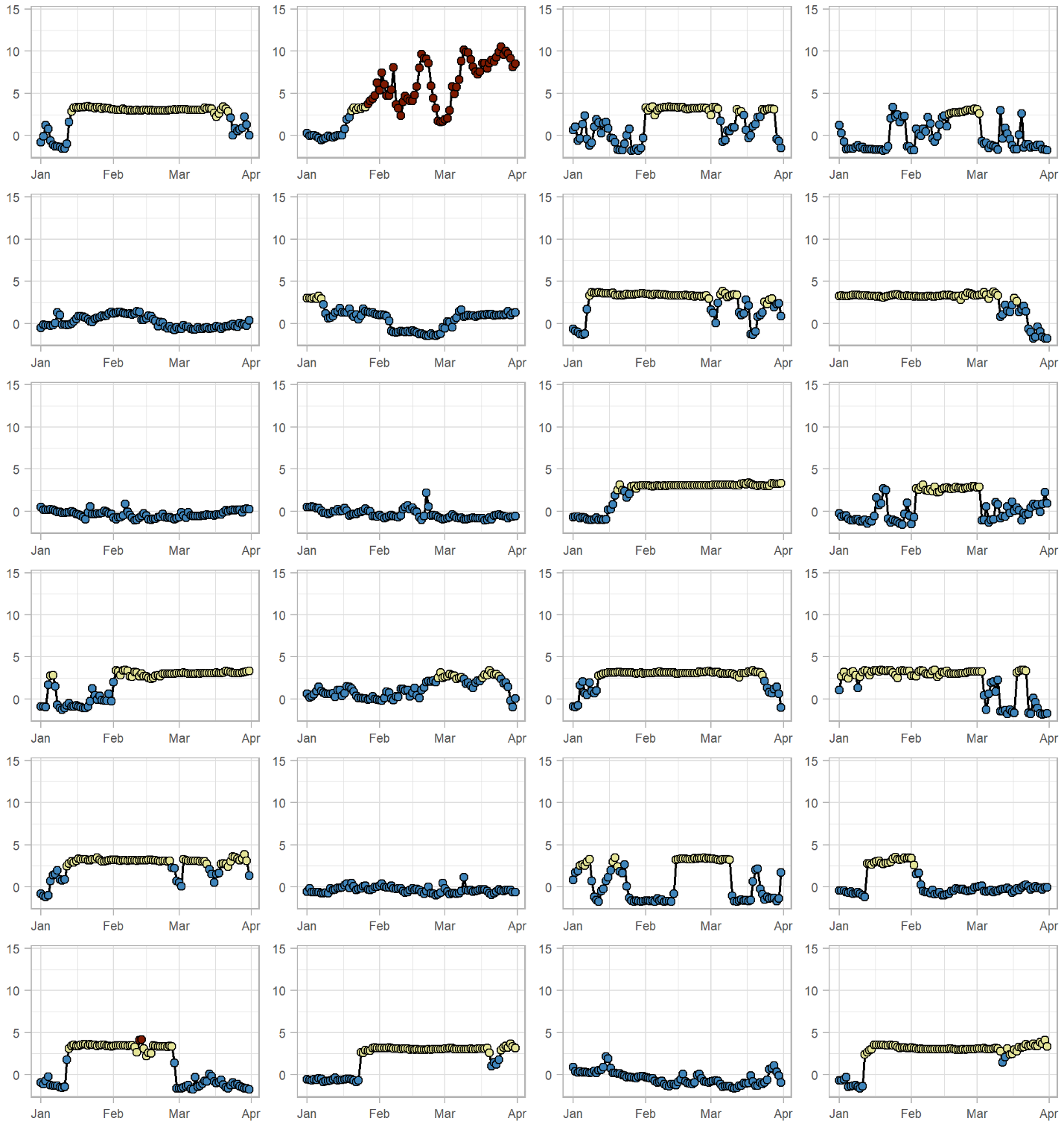


Figure S2. Plots of estimated sea surface temperature (SST) experienced during winter by each individual murre. Points are coloured according to the habitat state classification from a hidden Markov model with three states: Cold (blue), Warm (yellow), and Warmer (red).


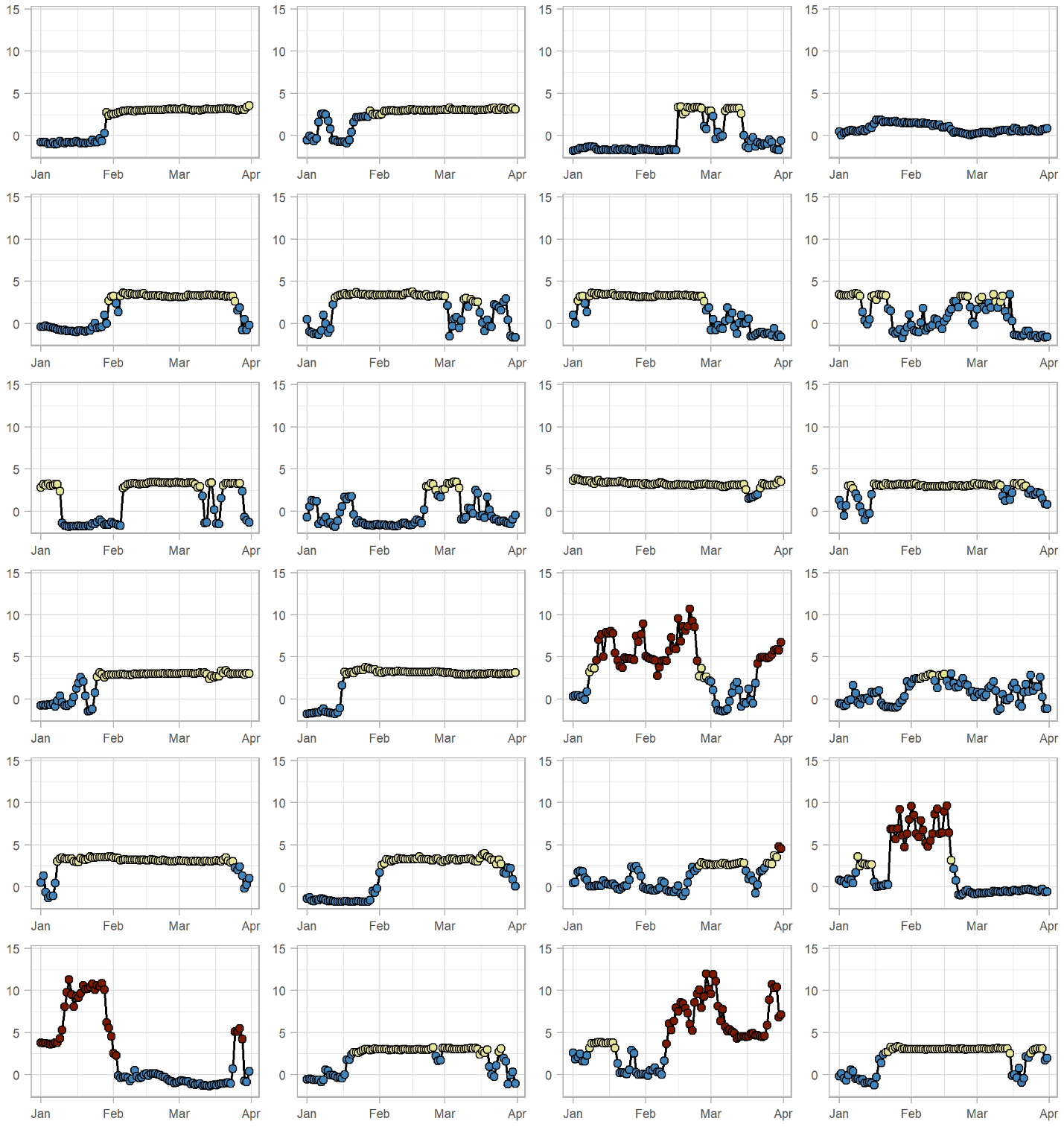
Figure S2 (cont). Plots of estimated sea surface temperature (SST) experienced during winter by each individual murre. Points are coloured according to the habitat state classification from a hidden Markov model with three states: Cold (blue), Warm (yellow), and Warmer (red).


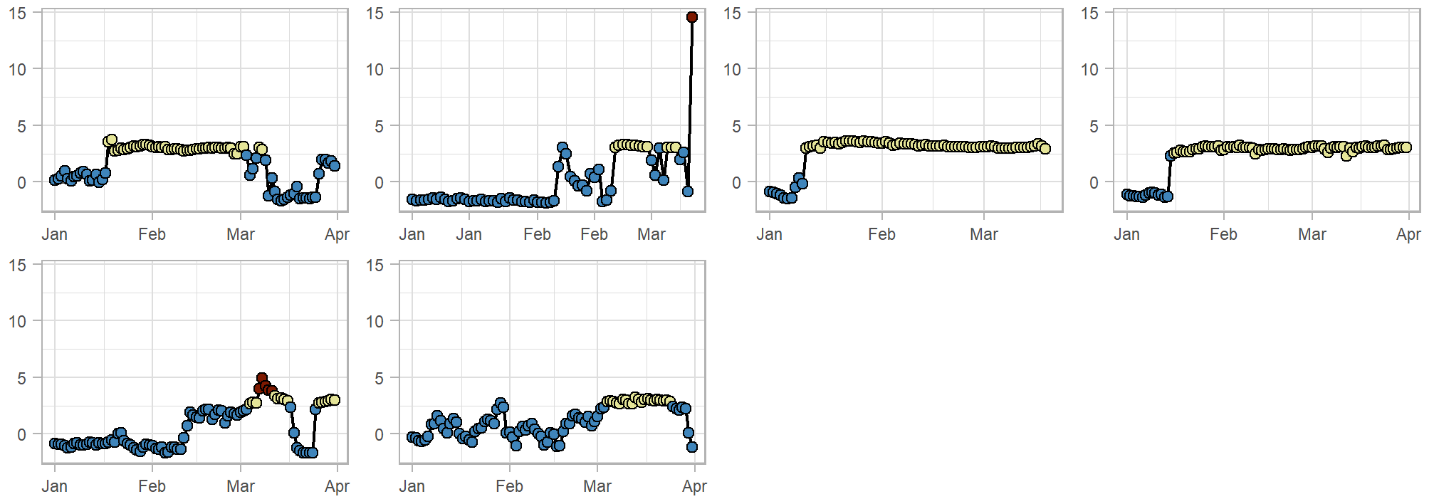


Figure S2 (cont). Plots of estimated sea surface temperature (SST) experienced during winter by each individual murre. Points are coloured according to the habitat state classification from a hidden Markov model with three states: Cold (blue), Warm (yellow), and Warmer (red).


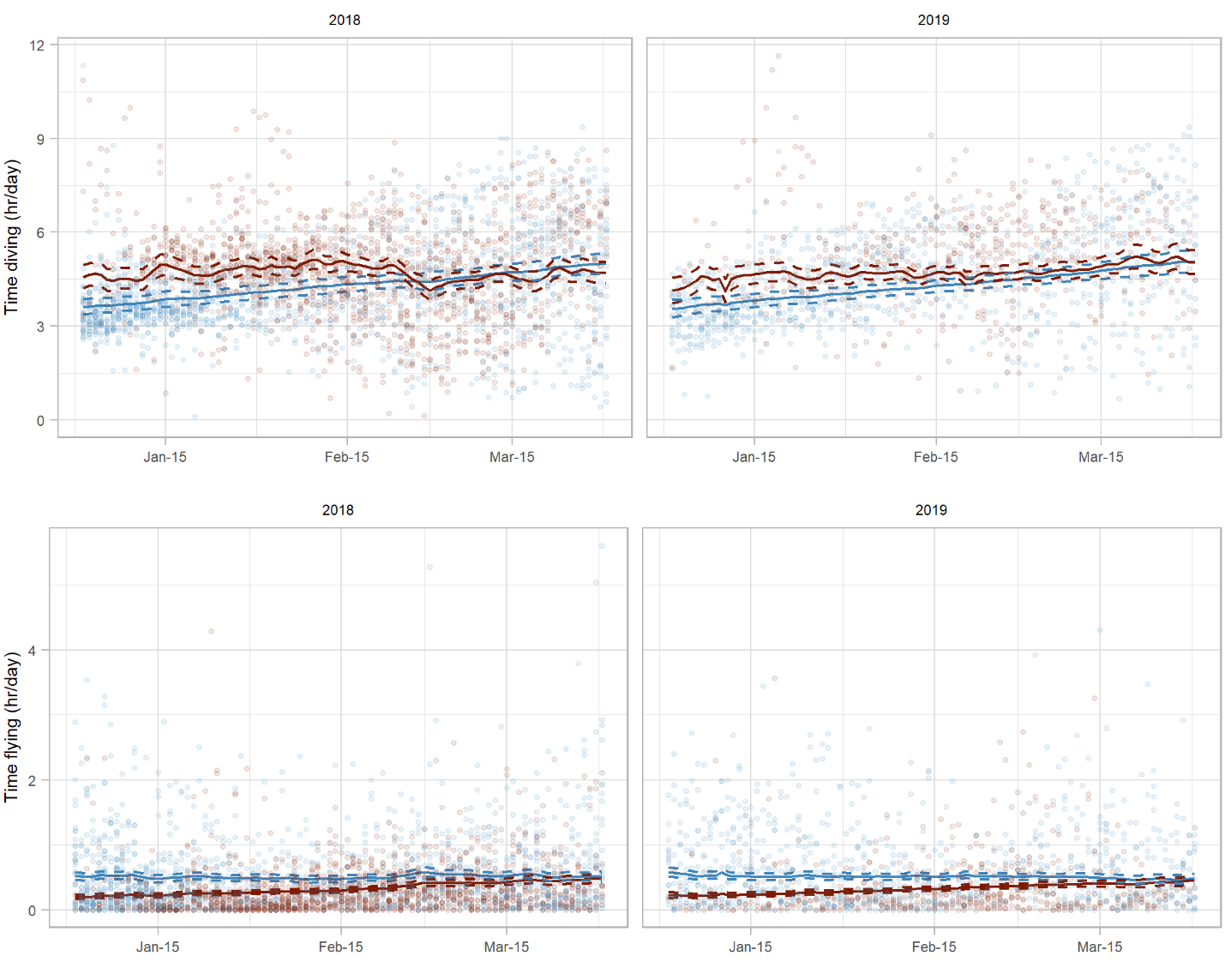


Figure S3. Time spent diving and flying for thick-billed murres in Cold water habitat (blue) and Warm water habitat (red) for 2018 and 2019, Lines show predicted values from generalized linear models that included fixed effects for habitat, day of year (DOY), and North Atlantic Oscillation (NAO), as well as two-way interactions between habitat and all other predictors. Solid lines are mean estimates and dashed lines are 95% confidence intervals. Points show the observed values for each individual each day. Note that y-axis ranges change among plots.

Table S2. AIC model comparison for generalized linear mixed effects models of daily time flying and diving. Predictor variables in model formulae are H = habitat, D = day of year, M = moon illumination, N = NAO. ZI refers to models that included zero-inflation parameters. All models included random effects for individual id (1|ID) and Ornstein-Uhlenbeck covariance structure to account for temporal autocorrelation. Column names refer to the number of parameters in each model (K), log-likelihood (LL), Akaike’s Information Criterion (AIC), delta AIC (∆AIC), and AIC weight. Only the ten models with the lowest AIC and the null model are shown.

| **Model** | **K** | **LL** | **AIC** | **∆AIC** | **wAIC** |
| --- | --- | --- | --- | --- | --- |
| ***Diving ~ H + D + N + H:D + H:N + (1\|ID)*** | ***10*** | ***6847*** | ***-13673.7*** | ***0.00*** | ***0.47*** |
| Diving ~ H + D + M + N + H:D + H:N + (1\|ID) | 11 | 6847 | -13672.8 | 0.91 | 0.30 |
| Diving ~ H + D + M + N + H:D + H:M + H:N + (1\|ID) | 12 | 6848 | -13672.1 | 1.62 | 0.21 |
| Diving ~ H + D + N + H:D + (1\|ID) | 9 | 6842 | -13666.9 | 6.87 | 0.02 |
| Diving ~ H + D + M + N + H:D + (1\|ID) | 10 | 6843 | -13665.6 | 8.12 | 0.01 |
| Diving ~ H + D + M + N + H:D + H:M + (1\|ID) | 11 | 6843 | -13664.3 | 9.43 | 0.00 |
| Diving ~ H + D + H:D + (1\|ID) | 8 | 6837 | -13657.2 | 16.59 | 0.00 |
| Diving ~ H + D + M + H:D + (1\|ID) | 9 | 6837 | -13655.3 | 18.43 | 0.00 |
| Diving ~ H + D + N + H:N + (1\|ID) | 9 | 6837 | -13655.2 | 18.50 | 0.00 |
| Diving ~ H + D + M + N + H:N + (1\|ID) | 10 | 6837 | -13654.3 | 19.45 | 0.00 |
| Diving ~ (1\|ID) | 5 | 6799 | -13588.9 | 84.80 | 0.00 |
| ***Flying ~ H + D + N + H:D + (1\|ID), ZI ~ H + H:D + (1\|ID)*** | ***16*** | ***10957*** | ***-21882.0*** | ***0.00*** | ***0.34*** |
| Flying ~ H + D + M + N + H:D + H:M + (1\|ID), ZI ~ H + H:D + (1\|ID) | 18 | 10959 | -21881.2 | 0.72 | 0.24 |
| Flying ~ H + D + N + H:D + H:N + (1\|ID), ZI ~ H + H:D + (1\|ID) | 17 | 10957 | -21880.0 | 1.98 | 0.13 |
| Flying ~ H + D + M + N + H:D + (1\|ID), ZI ~ H + H:D + (1\|ID) | 17 | 10957 | -21880.0 | 2.00 | 0.13 |
| Flying ~ H + D + M + N + H:D + H:M + H:N + (1\|ID), ZI ~ H + H:D + (1\|ID) | 19 | 10959 | -21879.2 | 2.72 | 0.09 |
| Flying ~ H + D + M + N + H:D + H:N + (1\|ID), ZI ~ H + H:D + (1\|ID) | 18 | 10957 | -21878.0 | 3.98 | 0.05 |
| Flying ~ H + D + H:D + (1\|ID), ZI ~ H + H:D + (1\|ID) | 15 | 10953 | -21876.0 | 5.97 | 0.02 |
| Flying ~ H + D + M + H:D + H:M + (1\|ID), ZI ~ H + H:D + (1\|ID) | 17 | 10954 | -21874.4 | 7.53 | 0.01 |
| Flying ~ H + D + M + H:D + (1\|ID), ZI ~ H + H:D + (1\|ID) | 16 | 10953 | -21874.1 | 7.90 | 0.01 |
| Flying ~ H + D + N + (1\|ID), ZI ~ H + H:D + (1\|ID) | 15 | 10942 | -21854.1 | 27.82 | 0.00 |
| Flying ~ (1\|ID), ZI ~ H + H:D + (1\|ID) | 12 | 10892 | -21760.5 | 121.49 | 0.00 |

Table S3. Parameter estimates for time spent diving and flying. Values are parameter estimates (± SE) from generalized linear mixed effects models. Conditional model parameters are reported on the log-link scale and zero-inflation parameters are reported on the logit-link scale. Sample size included 44 individual murres tracked for a combined 4302 days. Only parameters included in the most parsimonious models are shown (Table S2).

| **Model component** | **Parameter** | **Diving** | **Flying** |
| --- | --- | --- | --- |
| *Conditional* | (Intercept) | -1.742 (0.042)*** | -3.756 (0.061)*** |
|  | HabitatWarm | 0.253 (0.047)*** | -0.857 (0.090)*** |
|  | DOY | 0.005 (0.001)*** | -0.001 (0.001) |
|  | NAO | 0.013 (0.016) | -0.068 (0.024)** |
|  | DOY:HabitatWarm | -0.004 (0.001)*** | 0.009 (0.002)*** |
|  | NAO:HabitatWarm | 0.067 (0.022)** |  |
| *Zero-inflation* | (Intercept) |  | -3.733 (0.328)*** |
|  | HabitatWarm |  | 1.994 (0.375)*** |
|  | DOY |  | -0.001 (0.005) |
|  | HabitatWarm:DOY |  | -0.033 (0.008)*** |
| Significance: * <0.05, ** <0.01, *** <0.001 | | | |

Table S4. AIC model comparison for generalized linear mixed effects models of proportion of time diving during daylight, civil twilight, nautical twilight, and night. Predictor variables in model formulae are H = habitat, D = day of year, M = moon illumination, N = NAO. ZI refers to models that included zero-inflation parameters. All models included random effects for individual id (1|ID) and Ornstein-Uhlenbeck covariance structure to account for temporal autocorrelation. Column names refer to the number of parameters in each model (K), log-likelihood (LL), Akaike’s Information Criterion (AIC), delta AIC (∆AIC), and AIC weight. Only the ten models with the lowest AIC and the null model are shown.

| **Model** | **K** | **LL** | **AIC** | **∆AIC** | **wAIC** |
| --- | --- | --- | --- | --- | --- |
| ***Day ~ H + D + M + N + H:D + H:M + H:N + (1\|ID)*** | ***12*** | ***4228.9*** | ***-8433.8*** | ***0.00*** | ***0.80*** |
| Day ~ H + D + M + N + H:D + H:N + (1\|ID) | 11 | 4226.5 | -8430.9 | 2.88 | 0.19 |
| Day ~ H + D + M + N + H:D + H:M + (1\|ID) | 11 | 4223.4 | -8424.8 | 8.97 | 0.01 |
| Day ~ H + D + M + N + H:D + H:N + (1\|ID) | 10 | 4221.0 | -8422.0 | 11.77 | 0.00 |
| Day ~ H + D + M + N + H:M + H:N + (1\|ID) | 11 | 4221.7 | -8421.3 | 12.47 | 0.00 |
| Day ~ H + D + M + N + H:D + (1\|ID) | 10 | 4220.5 | -8420.9 | 12.88 | 0.00 |
| Day ~ H + D + M + N + H:N + (1\|ID) | 10 | 4219.2 | -8418.4 | 15.43 | 0.00 |
| Day ~ H + D + M + N + H:M + (1\|ID) | 10 | 4218.7 | -8417.4 | 16.42 | 0.00 |
| Day ~ H + D + M + N + (1\|ID) | 9 | 4215.8 | -8413.7 | 20.15 | 0.00 |
| Day ~ H + D + M + N + H:D + (1\|ID) | 9 | 4214.5 | -8410.9 | 22.86 | 0.00 |
| Day ~ (1\|ID) | 5 | 4140.5 | -8270.9 | 162.86 | 0.00 |
| ***Civil ~ H + D + M + H:M + (1\|ID)*** | ***9*** | ***6816.7*** | ***-13615.4*** | ***0.00*** | ***0.42*** |
| Civil ~ H + D + M + H:D + H:M + (1\|ID) | 10 | 6816.8 | -13613.7 | 1.73 | 0.18 |
| Civil ~ H + D + M + N + H:M + (1\|ID) | 10 | 6816.8 | -13613.5 | 1.89 | 0.16 |
| Civil ~ H + D + M + N + H:M + H:N + (1\|ID) | 11 | 6817.5 | -13612.9 | 2.46 | 0.12 |
| Civil ~ H + D + M + N + H:D + H:M + (1\|ID) | 11 | 6816.9 | -13611.7 | 3.65 | 0.07 |
| Civil ~ H + D + M + N + H:D + H:M + H:N + (1\|ID) | 12 | 6817.5 | -13611.0 | 4.40 | 0.05 |
| Civil ~ H + D + M + (1\|ID) | 8 | 6810.1 | -13604.2 | 11.17 | 0.00 |
| Civil ~ H + D + M + N + H:N + (1\|ID) | 10 | 6811.5 | -13603.0 | 12.43 | 0.00 |
| Civil ~ D + M + (1\|ID) | 7 | 6808.3 | -13602.6 | 12.80 | 0.00 |
| Civil ~ H + D + M + N + (1\|ID) | 9 | 6810.3 | -13602.6 | 12.84 | 0.00 |
| Civil ~ (1\|ID) | 5 | 6762.6 | -13515.2 | 100.23 | 0.00 |
| ***Nautical ~ H + D + M + N + H:D + H:M + H:N + (1\|ID), ZI ~ H + D + N + H:D + (1\|ID)*** | ***21*** | ***5445.8*** | ***-10849.7*** | ***0.00*** | ***0.74*** |
| Nautical ~ H + D + M + N + H:D + H:M + (1\|ID), ZI ~ H + D + N + H:D + (1\|ID) | 20 | 5443.5 | -10847.0 | 2.69 | 0.19 |
| Nautical ~ H + D + M + H:D + H:M + (1\|ID), ZI ~ H + D + N + H:D + (1\|ID) | 19 | 5440.6 | -10843.2 | 6.44 | 0.03 |
| Nautical ~ H + D + M + N + H:M + H:N + (1\|ID), ZI ~ H + D + N + H:D + (1\|ID) | 20 | 5441.3 | -10842.5 | 7.13 | 0.02 |
| Nautical ~ H + D + M + N + H:M + (1\|ID), ZI ~ H + D + N + H:D + (1\|ID) | 19 | 5440.0 | -10842.1 | 7.57 | 0.02 |
| Nautical ~ H + D + M + H:M + (1\|ID), ZI ~ H + D + N + H:D + (1\|ID) | 18 | 5437.9 | -10839.7 | 9.93 | 0.01 |
| Nautical ~ H + D + M + N + H:D + H:N + (1\|ID), ZI ~ H + D + N + H:D + (1\|ID) | 20 | 5436.0 | -10832.0 | 17.70 | 0.00 |
| Nautical ~ H + D + M + N + H:D + (1\|ID), ZI ~ H + D + N + H:D + (1\|ID) | 19 | 5434.4 | -10830.9 | 18.77 | 0.00 |
| Nautical ~ H + D + M + H:D + (1\|ID), ZI ~ H + D + N + H:D + (1\|ID) | 18 | 5432.5 | -10829.0 | 20.62 | 0.00 |
| Nautical ~ H + D + M + N + (1\|ID), ZI ~ H + D + N + H:D + (1\|ID) | 18 | 5431.2 | -10826.5 | 23.17 | 0.00 |
| Nautical ~ (1\|ID), ZI ~ H + D + N + H:D + (1\|ID) | 14 | 5331.1 | -10634.3 | 215.37 | 0.00 |
| ***Night ~ H + D + M + N + H:D + H:M + H:N + (1\|ID), ZI ~ H + D + M + N + H:D + (1\|ID)*** | ***19*** | ***353.7*** | ***-669.4*** | ***0.00*** | ***0.96*** |
| Night ~ H + D + M + N + H:M + H:N + (1\|ID), ZI ~ H + D + M + N + H:D + (1\|ID) | 18 | 349.4 | -662.9 | 6.59 | 0.04 |
| Night ~ H + D + M + N + H:D + (H:N + 1\|ID), ZI ~ H + D + M + N + H:D + (1\|ID) | 18 | 345.7 | -655.5 | 13.98 | 0.00 |
| Night ~ H + D + M + N + H:N + (1\|ID), ZI ~ H + D + M + N + H:D + (1\|ID) | 17 | 342.6 | -651.3 | 18.14 | 0.00 |
| Night ~ D + M + N + H:M + H:N + (1\|ID), ZI ~ H + D + M + N + H:D + (1\|ID) | 17 | 333.8 | -633.7 | 35.75 | 0.00 |
| Night ~ H + D + M + N + H:D + H:M + (1\|ID), ZI ~ H + D + M + N + H:D + (1\|ID) | 18 | 331.3 | -626.5 | 42.89 | 0.00 |
| Night ~ H + D + M + N + H:M + (1\|ID), ZI ~ H + D + M + N + H:D + (1\|ID) | 17 | 329.7 | -625.5 | 43.96 | 0.00 |
| Night ~ D + M + N + H:N + (1\|ID), ZI ~ H + D + M + N + H:D + (1\|ID) | 16 | 326.1 | -620.2 | 49.21 | 0.00 |
| Night ~ H + D + M + N + (1\|ID), ZI ~ H + D + M + N + H:D + (1\|ID) | 16 | 321.5 | -611.1 | 58.39 | 0.00 |
| Night ~ H + D + M + N + H:D + (1\|ID), ZI ~ H + D + M + N + H:D + (1\|ID) | 17 | 322.1 | -610.2 | 59.27 | 0.00 |
| Night ~ (1\|ID), ZI ~ H + D + M + N + H:D + (1\|ID) | 12 | 223.8 | -423.5 | 245.89 | 0.00 |

Table S5. Parameter estimates for time proportion of time diving during daylight, civil twilight, nautical twilight, and night.Values are parameter estimates (± SE) from generalized linear mixed effects models. Conditional model parameters are reported on the log-link scale and zero-inflation parameters are reported on the logit-link scale. Sample size included 44 individual murres tracked for a combined 4302 days. Only parameters included in the most parsimonious models are shown (Table S4).

| **Parameter** | **Day** | **Civil** | **Nautical** | **Night** |
| --- | --- | --- | --- | --- |
| *Conditional terms* |  |  |  |  |
| (Intercept) | 1.345 (0.106)*** | -1.659 (0.064)*** | -3.079 (0.097)*** | -2.264 (0.129)*** |
| HabitatWarm | -0.868 (0.138)*** | 0.192 (0.049)*** | 1.344 (0.127)*** | 0.759 (0.168)*** |
| DOY | 1.047 (0.128)*** | -0.985 (0.089)*** | -0.668 (0.126)*** | -0.294 (0.185) |
| Moon | -0.141 (0.102) | 0.021 (0.052) | -0.045 (0.091) | 0.159 (0.152) |
| NAO | 0.081 (0.044) |  | -0.007 (0.044) | 0.086 (0.079) |
| DOY:HabitatWarm | 0.708 (0.185)*** |  | -0.525 (0.173)** | -0.744 (0.253)** |
| Moon:HabitatWarm | -0.296 (0.134)* | -0.263 (0.072)*** | -0.532 (0.118)*** | 0.729 (0.181)*** |
| NAO:HabitatWarm | 0.208 (0.062)*** |  | -0.122 (0.056)* | -0.614 (0.093)*** |
| *Zero-inflation terms* |  |  |  |  |
| (Intercept) |  |  | -1.743 (0.355)*** | 2.807 (0.387)*** |
| HabitatWarm |  |  | -3.594 (0.597)*** | -3.735 (0.446)*** |
| DOY |  |  | 0.082 (0.488) | 1.563 (0.508)** |
| Moon |  |  |  | -1.802 (0.255)*** |
| NAO |  |  | 0.089 (0.153) | 0.343 (0.126)** |
| HabitatWarm:DOY |  |  | 2.554 (0.866)** | 2.952 (0.705)*** |
| HabitatWarm:NAO |  |  | -0.690 (0.262)** |  |
| Significance: * <0.05, ** <0.01, *** <0.001 | | | | |


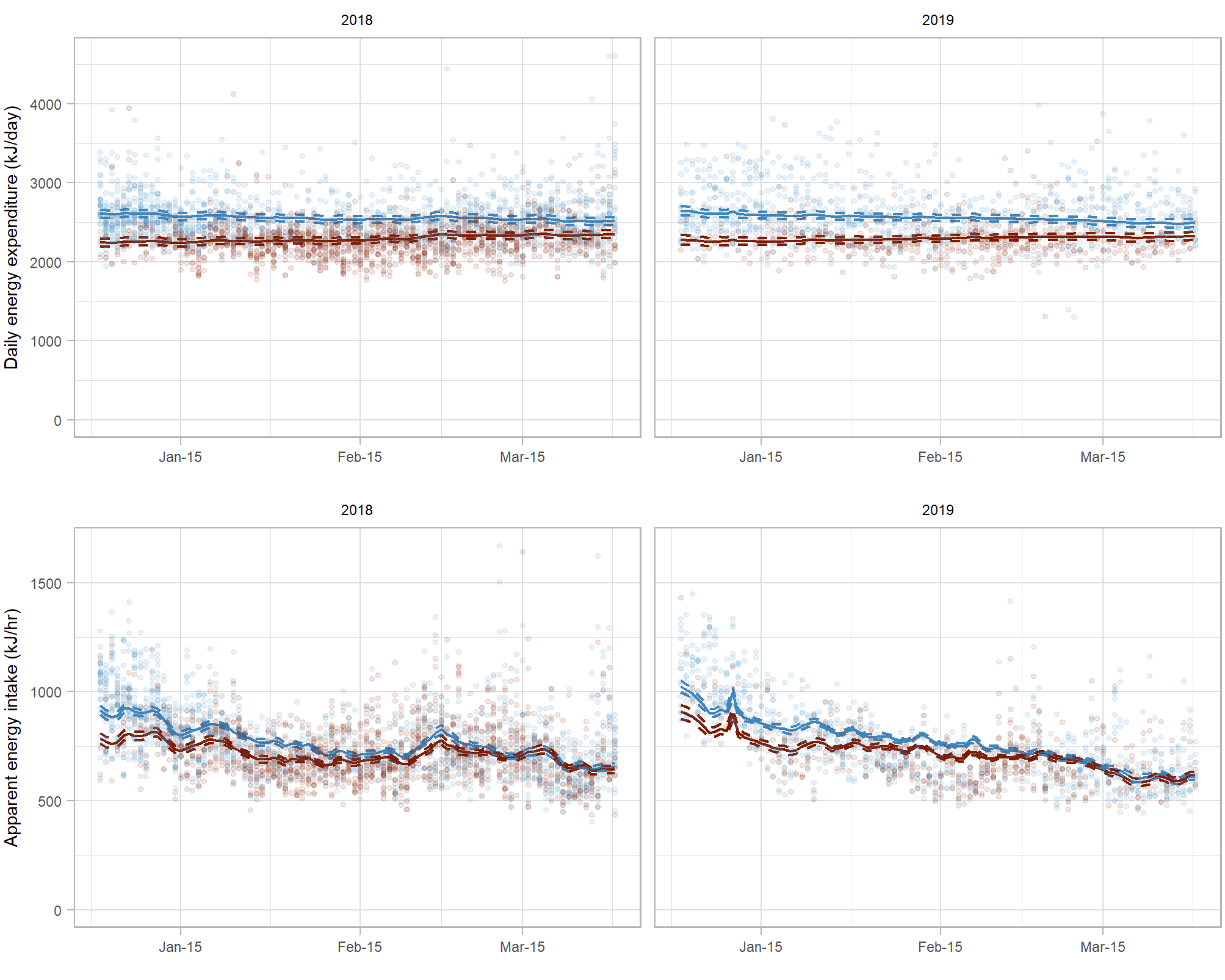


Figure S4. Daily energy expenditure (kJ/day) and apparent energy expenditure (kJ/hour) of thick-billed murres in Cold water habitat (blue) and Warm water habitat (red) for 2018 and 2019. Lines show predicted values from generalized linear models that included fixed effects for habitat, day of year (DOY), moon, and North Atlantic Oscillation (NAO), as well as two-way interactions between habitat and all other predictors. Solid lines are mean estimates and dashed lines are 95% confidence intervals. Points show the observed values for each individual each day. Note that y-axis ranges change among rows.

Table S6. AIC model comparison for generalized linear mixed effects models of daily energy expenditure (DEE) and apparent energy intake (AEI). Predictor variables in model formulae are H = habitat, D = day of year, M = moon illumination, N = NAO). All models included random effects for individual id (1|ID) and Ornstein-Uhlenbeck covariance structure to account for temporal autocorrelation. Column names refer to the number of parameters in each model (K), log-likelihood (LL), Akaike’s Information Criterion (AIC), delta AIC (∆AIC), and AIC weight. . Only the ten models with the lowest AIC and the null model are shown.

| **Model** | **K** | **LL** | **AIC** | **∆AIC** | **wAIC** |
| --- | --- | --- | --- | --- | --- |
| DEE ~ H + D + M +N + H:D + H:M + (1\|ID) | 11 | -29404 | 58829.9 | 0.00 | 0.29 |
| ***DEE ~ H + D + N + H:D + (1\|ID)*** | ***9*** | ***-29406*** | ***58830.0*** | ***0.13*** | ***0.27*** |
| DEE ~ H + D + M +N + H:D + H:M + H:N + (1\|ID) | 12 | -29404 | 58831.6 | 1.70 | 0.12 |
| DEE ~ H + D + M +N + H:D + (1\|ID) | 10 | -29406 | 58831.6 | 1.75 | 0.12 |
| DEE ~ H + D + N + H:D + H:N + (1\|ID) | 10 | -29406 | 58831.8 | 1.96 | 0.11 |
| DEE ~ H + D + M +N + H:D + H:N + (1\|ID) | 11 | -29406 | 58833.5 | 3.61 | 0.05 |
| DEE ~ H + D + H:D + (1\|ID) | 8 | -29410 | 58835.0 | 5.17 | 0.02 |
| DEE ~ H + D + M + H:D + H:M + (1\|ID) | 10 | -29408 | 58835.3 | 5.38 | 0.02 |
| DEE ~ H + D + M + H:D + (1\|ID) | 9 | -29409 | 58836.3 | 6.39 | 0.01 |
| DEE ~ H + D + M +N + H:M + (H:N + 1\|ID) | 11 | -29414 | 58849.6 | 19.68 | 0.00 |
| DEE ~ (1\|ID) | 5 | -29541 | 59093.0 | 263.11 | 0.00 |
| ***AEI ~ H + D + M + N + H:D + H:M + (1\|ID)*** | ***11*** | ***-26827*** | ***53675.5*** | ***0.00*** | ***0.41*** |
| AEI ~ H + D + M + N + H:D + H:M + H:N + (1\|ID) | 12 | -26826 | 53675.9 | 0.41 | 0.33 |
| AEI ~ H + D + M + N + H:D + (1\|ID) | 10 | -26829 | 53677.7 | 2.15 | 0.14 |
| AEI ~ H + D + M + N + H:D + H:N + (1\|ID) | 11 | -26828 | 53678.6 | 3.05 | 0.09 |
| AEI ~ H + D + N + H:D + (1\|ID) | 9 | -26832 | 53681.1 | 5.61 | 0.02 |
| AEI ~ H + D + N + H:D + H:N + (1\|ID) | 10 | -26831 | 53682.3 | 6.75 | 0.01 |
| AEI ~ H + D + M + N + H:D + H:M + H:N + (1\|ID) | 11 | -26836 | 53693.1 | 17.55 | 0.00 |
| AEI ~ H + D + M + N + H:D + H:N + (1\|ID) | 10 | -26838 | 53695.8 | 20.24 | 0.00 |
| AEI ~ H + D + M + N + H:D + H:M + (1\|ID) | 10 | -26839 | 53698.2 | 22.69 | 0.00 |
| AEI ~ H + D + N + H:D + H:N + (1\|ID) | 9 | -26841 | 53699.3 | 23.74 | 0.00 |
| AEI ~ (1\|ID) | 5 | -27019 | 54048.1 | 372.61 | 0.00 |

Table S7. Parameter estimates for daily energy expenditure (DEE) and apparent energy expenditure (AEI). Values are parameter estimates (± SE), shown on the log-link scale, from generalized linear mixed effects models with Gamma distribution. Only parameters included in the most parsimonious models are shown (Table S6).

| **Parameter** | **DEE** | **AEI** |
| --- | --- | --- |
| (Intercept) | 7.8728 (0.0098)*** | 6.8647 (0.0116)*** |
| HabitatWarm | -0.1520 (0.0113)*** | -0.1136 (0.0186)*** |
| DOY | -0.0448 (0.0113)*** | -0.3858 (0.0165)*** |
| Moon |  | -0.0073 (0.0138) |
| NAO | -0.0086 (0.0032)** | -0.0692 (0.0052)*** |
| DOY:HabitatWarm | 0.0849 (0.0179)*** | 0.1374 (0.0272)*** |
| Moon:HabitatWarm |  | -0.0404 (0.0198)* |
| Significance: * <0.05, ** <0.01, *** <0.001 | | |

# References

1. Lisovski S, Wotherspoon S, Sumner M. TwGeos: Basic data processing for light-level geolocation archival tags. 2016.

2. Linnebjerg JF, Huffeldt NP, Falk K, Merkel FR, Mosbech A, Frederiksen M. Inferring seabird activity budgets from leg-mounted time–depth recorders. J Ornithol. 2014;155:301–6.

3. Halpin LR, Ross JD, Ramos R, Mott R, Carlile N, Golding N, et al. Double-tagging scores of seabirds reveals that light-level geolocator accuracy is limited by species idiosyncrasies and equatorial solar profiles. Methods in Ecology and Evolution. 2021;12:2243–55.

4. Merkel B, Phillips RA, Descamps S, Yoccoz NG, Moe B, Strøm H. A probabilistic algorithm to process geolocation data. Mov Ecol. 2016;4:26.

5. Reynolds RW, Smith TM, Liu C, Chelton DB, Casey KS, Schlax MG. Daily high-resolution-blended analyses for sea surface temperature. Journal of Climate. 2007;20:5473–96.
